# Novel Matrix Proteins of *Pteria penguin* Pearl Oyster Shell Nacre Homologous to the Jacalin-Related β-Prism Fold Lectins

**DOI:** 10.1371/journal.pone.0112326

**Published:** 2014-11-06

**Authors:** Takako Naganuma, Wataru Hoshino, Yukihiro Shikanai, Rie Sato, Kaiyue Liu, Saho Sato, Koji Muramoto, Makoto Osada, Kyosuke Yoshimi, Tomohisa Ogawa

**Affiliations:** 1 The Department of Biomolecular Sciences, Graduate School of Life Sciences, Tohoku University, Sendai 980-8577, Japan; 2 Center for Interdisciplinary Research, Tohoku University, Sendai 980-8578, Japan; 3 Graduate School of Agricultural Science, Tohoku University, Sendai 981-8555, Japan; 4 Graduate School of Environmental Studies, Tohoku University, Sendai 980-8578, Japan; Laboratoire de Biologie du Développement de Villefranche-sur-Mer, France

## Abstract

Nacreous layers of pearl oyster are one of the major functional biominerals. By participating in organic compound-crystal interactions, they assemble into consecutive mineral lamellae-like photonic crystals. Their biomineralization mechanisms are controlled by macromolecules; however, they are largely unknown. Here, we report two novel lectins termed PPL2A and PPL2B, which were isolated from the mantle and the secreted fluid of *Pteria penguin* oyster. PPL2A is a hetero-dimer composed of α and γ subunits, and PPL2B is a homo-dimer of β subunit, all of which surprisingly shared sequence homology with the jacalin-related plant lectin. On the basis of knockdown experiments at the larval stage, the identification of PPLs in the shell matrix, and *in vitro* CaCO_3_ crystallization analysis, we conclude that two novel jacalin-related lectins participate in the biomineralization of *P. penguin* nacre as matrix proteins. Furthermore, it was found that trehalose, which is specific recognizing carbohydrates for PPL2A and is abundant in the secreted fluid of *P. penguin* mantle, functions as a regulatory factor for biomineralization via PPL2A. These observations highlight the unique functions, diversity and molecular evolution of this lectin family involved in the mollusk shell formation.

## Introduction

Biomineralization is a process of selective extraction of metal ions into specific functional structures under strict biological control [1]–[4]. The activity is required for the formation of bone, teeth, eggshells, coral reefs, marine phytoplankton, and microlens of brittlestars and shells. The biominerals are natural nano-composites of protein-crystal interactions, and the molecular mechanisms underlying the biomineral formation have inspired nanotechnology applications by virtue of their bottom-up approach [5], [6]. Calcium salts and silicate are widely used in various organisms. In particular, three types of calcium carbonate structures (calcite, aragonite, and vaterite) are major materials used in marine biominerals [7]. Interestingly, mother of pearl shells can concurrently produce two different crystal types, calcite and aragonite. Nacreous layers of pearl oyster are composed of aragonite tablets, which are arranged in consecutive mineral lamellae like a photonic crystal. Recently, several matrix proteins have been identified from various layers of mollusk lamellae. In the nacreous (aragonite) layer, these include lustrin A [8], MSI60 [9], N16 [10], pearlin [11], N14 [12], perlucin [13], AP7 and AP24 [14], Pif [15], and PfN23b [16]. Additionally, p12 [17], MSI3 [9], aspein [18], and prismalin-14 [19] have been identified in the calcite layer. It is known that the major components of the matrix are polysaccharide β-chitin, a relatively hydrophobic silk-like proteins and a complex of hydrophilic proteins, many of which are rich in aspartic acid and very acidic, and amorphous precursor phase [20], [21]. Especially, the acidic proteins, which are assumed to be β-sheet conformation in the presence of calcium ion, have been noted in the biomineralization processes due to their unique distribution in nacre [22]. However, the detailed molecular mechanisms of biomineralization still remain to be mysterious.

Lectins, which are carbohydrate recognition proteins, have been found in viruses, bacteria, fungi, plants, and animals. They agglutinate cells and/or precipitate glycoconjugates *via* specific interactions with sugar chains [25]. To date, numerous lectins have been isolated from animals, and classified on the basis of their CRD sequences. Classes include the C-type lectins (CTL), galectins, P-type, I-type (siglecs), F-type (pentraxins), tachylectin-2 and RBLs [24], [26], [27].

Previously, we isolated an 18 kDa lectin, (which we now refer to as PPL1) from the mantle of *Pteria penguin* large-winged pearl shells, and determined its primary structure and molecular properties [23]. PPL1 has two tandem carbohydrate recognition domain (CRD) structures in its sequence, which has sequence homology with rhamnose-binding lectins (RBLs) from various fish eggs, and a galactose-binding lectin (SUEL) from sea urchin eggs [24]. PPL1 recognizes D-galactose, methyl-D-galactopyranoside, and *N*-acetyl-D-lactosamine, and also has a strong agglutinating activity against the marine bacteria Aeromonas salmonicidae, indicating that PPL1 may act as a biodefense molecule in *P. penguin*. In addition to PPL1, the unadsorbed fraction of the mucin-Sepharose 4B column chromatography retained strong hemagglutination activity, indicating that other novel lectin(s) existed in the mantle and its fluid.

The secreted fluid from the outer mantle epithelium of bivalves are believed to contain various components of the shell matrix including proteins, glycoproteins, and polysaccharides, together with the mineral precursors required during the calcification process [28]. This raised the possibility that lectins from the secreted fluid of *P. penguin* could be involved not only in biodefense but also in shell formation. In the present study, two jacalin-related lectins named PPL2A and PPL2B were isolated from the mantle and secreted fluid of *P. penguin*. Characterization of their structures and biochemical activities revealed that these PPL2s are matrix substrates required for the process of biomineralization.

## Materials and Methods

### Materials

Cultured large winged pearl shells (*Pteria penguin*, 6-years-old) and their larva were kindly provided by Amami South Sea & Mabe pearl Co. Ltd. (former Amami-branch, Tasaki & Co. Ltd.), Kagoshima, Japan. The mantle was collected and stored at −80°C until use. Resource S column, Sephadex G-15 and Sepharose 4B gels were purchased from GE Healthcare (NJ, USA). TSKgel sugar AXI, TSKgel ODS 120T, and TSKgel Amide 80 columns were from Tosoh (Tokyo, Japan). Trehalose-Sepharose 4B gel was prepared according to the method of Matsumoto *et al*. [29]. Mucin type I from bovine submaxillary glands and mucin type II from porcine stomach were purchased from Sigma Chemical (St. Louis, MO, USA). *Achromobacter* protease I and *Staphylococcus aureus* V8 protease were purchased from Wako Pure Chemical (Osaka, Japan). Glycopeptidase A was purchased from Seikagaku Kogyo (Tokyo, Japan). Trehalose was purchased from Hayashibara (Okayama, Japan). All the other reagents were of the purest grade commercially available.

### Scanning electron microscopy

The shells were randomly cut into pieces of 5×5 mm^2^ by using a low speed wheel cutter (Model 650, South Bay Tech. Inc.) with a diamond blade (φ101.6×0.3t×φ12.7, Refinetec Co. Ltd, Yokohama, Japan), and each piece was ground using a sanding machine (RotoPol-25, Marumoto Struers, Japan) to remove the outer shell part (calcite). Surface microstructures of the nacreous layer were observed using a scanning electron microscope (SEM) with SEM S-4300E or S-4200 (Hitachi, High-Tech. Co., Japan) after coating with osmium tetroxide or platinum-palladium for 1 s or 150 s, respectively, using Neo Osmium coater Neoc-ST (Meiwafosis, Co., Osaka, Japan).

### Isolation of *Pteria penguin* lectin 2 (PPL2)

The mantle was homogenized with 50 mM Tris-HCl (pH 7.5) containing 0.5 M NaCl and 5 mM CaCl_2_ (TBS). After centrifuged at 23,000×g for 20 min at 4°C, the supernatant was subjected to affinity chromatography on mucin-Sepharose 4B. The adsorbed fraction gave PPL1, of which sequence and biological properties have been previously reported [23]. Since the unadsorbed fraction retained strong hemagglutination activity that inhibited by trehalose, unadsorbed substances were purified by trehalose-Sepharose 4B affinity column. Adsorbed proteins were further purified by cation exchange chromatography on a Resource S column in 20 mM 2-mopholinoethansulfonic acid (MES) buffer (pH 7.0) using a linear gradient of 0 to 2 M NaCl.

### Two-dimensional polyacrylamide gel electrophoresis and SDS-PAGE

The proteins were isoelectrofocussed using the IPGphor, isoelectric focusing (IEF) system (Amersham) with immobilized pH gradient (IPG) strips with pH 3–10 or 3-11NL (11 cm length each) according to the manufacturer’s directions. For IPG strips of 3-11NL, the protein sample was rehydrated in 8 M urea, 2 M thiourea, 4% CHAPS, 40 mM DTT, and 0.5% IPG buffer pH 3-11NL. IEF was carried out with the following program: 500 V constant for 1 h, gradient to 1000 V for 1 h, gradient to 6000 V within 2 h then 6000 V for 50 min for a total of 20000 Vh.

The strip was then subjected to sodium dodecyl sulfate polyacrylamide gel electrophoresis (SDS-PAGE) for 2D-PAGE. SDS-PAGE was performed on 15% slab gels according to the method of Laemmli in the presence or absence of 2-mercaptoethanol, and protein bands were stained using Coomassie Brilliant Blue R-250 or silver staining kit (Wako Pure Chemical., Osaka, Japan).

### Hemagglutination assay and inhibition assay

Samples were serially diluted with 50 µl of 0.15 M NaCl on microtiter plates and mixed with equal volume of 2% rabbit erythrocyte suspension for 1 h. The hemagglutination activity was defined as the titer value of maximum dilution with positive agglutination of 2% rabbit erythrocytes. The inhibitory effects of saccharides on hemagglutination were also assayed. Briefly, the saccharide solutions (25 µl) were diluted 2-fold in series on microtiter plates and incubated with 25 µl of the lectin solution having hemagglutination titer values of 2^−3^ for 15 min. The rabbit erythrocytes suspension (2%, 50 µl) was added to the mixture and incubated for another 30 min. The inhibitory activities were estimated by the minimum concentration of sugar needed to cause negative hemagglutination.

### Identification of free sugars in *Pteria penguin* organs

To identify sugars in the mantle, adductor muscle, gill and body fluid, free sugars and polyols were analyzed by silica-gel thin layer chromatography (TLC). The mantle, adductor muscle and gill were homogenized in 10 mM Tris-HCl buffer (pH 7.5) containing 0.5 M NaCl and 5 mM CaCl_2_, respectively. After centrifuged at 8,000×g for 30 min at 4°C, the supernatant and body fluid were lyophilized. Each sample was dissolved in 300 µl of ethanol, and heated at 90°C for 10 min, bath-sonicated for 15 min, and centrifuged at 5000×g for 3 min. This extraction steps were repeated twice. The supernatants were pooled, frozen at −80°C and freeze-dried. Each sample was re-suspended in 10 µl of 50% ethanol with vortexing. Five microliters of each suspension was loaded on a thin layer plate (Silica Gel 60, Merck). Samples and standards were developed by ethyl acetate/acetic acid/methanol/water (60∶15∶15∶10), and were visualized by the orcinol-sulfuric acid method [30].

### Amino acid sequencing and cDNA sequencing

Purified PPL2A and PPL2B were reduced by dithiothreitol, *S*-carboxamidomethylated, and digested using endoproteinase *Achromobactor* protease I (substrate/enzyme (S/E), 100∶1), endoproteinase Arg-C (S/E, 100∶1), and *S. aureus* V8 protease (S/E, 50∶1), respectively. Each digest was separated by reversed-phase HPLC on a TSKgel ODS 120T column (4.6×250 mm) using a linear gradient of acetonitrile in 0.1% trifluoroacetic acid (TFA). The amino acid sequences of isolated proteins and peptides were determined by a gas-phase protein sequencer (PPSQ-10; Shimazu, Kyoto, Japan).

The positions of the disulfide bonds in PPL2 subunits were identified by analyzing the peptide fragments derived from the unmodified proteins upon endoproteinase Lys-C cleavage. The amino acid sequences of isolated peptide fragments were determined by the combined use of a protein sequencer and MALDI-TOF mass spectrometer.

Total RNA was extracted from the mantle of *P. penguin* by the guanidium thiocyanate/phenol/chloroform method, and poly (A)+ RNA was purified using the Micro-Fast Track mRNA Isolation kit (Invitrogen, Carlsbad, Calif.) as previously reported [23]. A cDNA library was constructed from poly (A)+ RNA using the Marathon cDNA Amplification kit (Clontech, Palo Alto, Calif., USA). To amplify the cDNAs of PPL2A and PPL2B, we designed the sense and anti-sense gene-specific primers (GSPs) based on the partial amino acid sequences of PPL2A and PPL2B, respectively (Table S1 in File S1). PCR was performed with KOD polymerase (Toyobo Co., Tokyo, Japan) for 30 cycles of repeating denaturation at 96°C for 30 sec, annealing at 45°C for 30 sec and extension at 68°C for 1 min using cDNA library as a template and primers. Full-length cDNA sequences of PPL2 subunits were determined by 3′- and 5′- rapid amplification of cDNA ends (RACE) with GSP for each PPL2 subunit (Table S1 in File S1) and adaptor primer 1 (AP1), 5′-CCATCCTAATACGACTCACTATAGGGC-3′. Each amplified cDNA fragment was subcloned into a pCR–Blunt II-TOPO plasmid (Invitrogen), and the nucleotide sequence was determined by Applied Biosystems Model 377 or 310 DNA sequencers. The nucleotide sequences of cDNAs encoding PPL2α, PPL2β, and PPL2γ subunits were deposited in Genbank database under accession numbers AB425237, AB425238, and AB425239, respectively.

Sequences were searched by BLAST program. The amino acid sequences of PPL2A (α and γ subunits) and PPL2B (β subunit) were compared with other jacalin-related lectins using a multiple alignment computed by Clustal W program [31]. A total 10 CRDs of jacalin-related lectins were retrieved from the NCBI/DDBJ/EMBL database. The source of the sequences used in the structural comparison are as follows; Jacalin: Galactose-binding lectin from *A. integer* (AAA32677); MPA: *Maclura pomifera* lectin (P18674); banana-JRL: *Musa acuminata* lectin (ABS86034); CCA: *Castanea crenata* lectin (P82859); heltuba: *Helianthus tuberosus* lectin (AAD11575); GRFIN: *Griffithsia* sp. lectin griffithsin (P84801); mouse-ZG16: mouse zymogen granule protein 16 (NP081194); rat-ZG16 (BAC24023); human-ZG16 (BAC20361). A phylogenetic tree was constructed by the improved neighbor-joining algorithm, BIONJ [32] using the PhyML (ver 3.1) program [33] in SeaView package (ver. 4.5.3) [34] after the aligned sequences of mature protein regions of JRLs were treated with Gblocks program [35]. The degrees of confidence for internal lineages in phylogenetic tree were determined by the bootstrap procedure [36] with 1,000 replicates in SeqView package.

### Purification and dephosphorylation of matrix proteins from nacreous layer

After removed the outer calcite layer by grinding, the nacreous layer pieces were decalcified by soaking in 1 M acetic acid for 4 to 6 days at RT with gentle stirring, and the matrix proteins were extracted with 70% formic acid for 2 days at 37°C.

For 2D PAGE analysis, the 70% formic acid-extracted nacreous matrix proteins were precipitated by acetone, and dissolved in 2D electrophoresis sample rehydration buffer (5 M urea with 2 M thiourea, 0.5% NP40 and 20 mM DTT). Dephosphorylation of nacreous matrix proteins was conducted by incubating with bacteria alkaline phosphatase (BAP, 0.6 U) at 30°C overnight in 50 mM Tris-HCl buffer (pH 9.0) containing 1 mM MgCl_2_. Furthermore, to identify the matrix proteins from nacreous layer, nanoLC-MALDI TOF MS/MS analysis was achieved for urea-extracted and 70% formic acid-extracted matrix proteins, respectively. The extracted proteins were concentrated by acetone precipitation, and reduced by TCEP, alkylated, and digested with trypsin (Wako Pure Chemical., Osaka, Japan) at 37°C overnight. Then, the resulting peptide pools were separated by nano LC system, and spotted onto MALDI target plates after desalted by using Sep-Pak cartridge column. The molecular mass and sequence of peptides on MALDI target plates were analyzed as described above.

### Preparation and characterization of antisera against PPL2s and PPL1

Anti-PPL2A and anti-PPL2B antisera were raised in mice (ICR-mice, 17–19 g, 4 weeks old, males) by repeated intraperitoneal injections of purified PPL2A and PPL2B (100 µg each) emulsified at a 1∶1 v/v ratio with Freund’s complete adjuvant (initial immunization only; Pierce Biotechnology, Rockford, IL), respectively. The specificities of antibodies were checked by dot blot and Western blot analyses. After four booster injections of PPL2A and PPL2B at >3-week intervals, respectively, antisera were collected and stored at −80**°**C. The specificities of antibodies were checked by dot blot and Western blot analyses. Finally, anti-PPL2A antibody, which shows the antigen specificities against PPL2A α and γ subunits and also PPL2B β subunit, was obtained. Anti-PPL1 antibody was prepared using purified PPL1 as antigens.

Furthermore, to obtain more specific antibodies against each subunit, PPL2α, PPL2β, and PPL2γ, polyclonal antibodies were obtained from Medical Biology Laboratory (MBL) Co. (Japan). Antibodies were prepared by immunization of rabbits with the synthetic peptide epitope-KLH conjugates, of which sequence were predicted with the high-antigenicity as follows; CSQYLTKVGGNGGGA for PPL2α(3–17), LKGGAFTDKSKANNGDIC for PPL2β(14–30), and DNWWKRGSMKEYTLGAC for PPL2γ(64–79), respectively. The specificities and sensitivities of antibodies were checked by ELISA (data not shown) and Western blotting analyses.

### Western blot analysis and immunohistochemistry

For Western blot analysis, proteins separated by SDS-PAGE were transferred onto polyvinylidene fluoride (PVDF) membranes under semidry conditions with iBlot Gel Transfer system (Invitrogen). The blotted PVDF was blocked with 1% bovine serum albumin (BSA) in phosphate-buffered saline (PBS) containing 0.1% Tween 20 (TPBS) overnight at 4°C. After washing 3 times with TPBS, the membrane was incubated with anti-PPL1 and anti-PPL2s mouse IgGs, respectively. Anti-PPL2α(3–17), anti-PPL2β(14–30), and anti-PPL2γ(64–79) rabbit polyclonal antibodies were also used. Each PVDF membrane was washed 3 times with TPBS, and then membranes were incubated with horseradish peroxidase (HRP)-conjugated anti-mouse goat IgG or HRP-conjugated anti-rabbit IgG. The detection was carried out with 0.2% of 3, 3′-diaminobenzidine (DAB) for HRP or ECL Plus (Amersham) for chemiluminescence method. The chemiluminescence images were directly acquired in a chemiluminescent imaging system equipped with highly-cooled CCD cameras, AE9300-Ez-Capture MG, and analyzed by densitograph software, CS Analyzer (ver 3.0) (ATTO, Tokyo, Japan).

The pieces of nacreous layer were partially demineralized by incubating with 10 ml cation-exchange resin (Dowex 50×8) in 4% formaldehyde solution containing 0.5% cetylpyridinium chloride (CPC) in a sealed dialysis membrane (3,500 MW cut-off). After decalcification, the pieces were washed with distilled water, and then were suspended with 1% BSA in TPBS overnight at 4°C. After washing 3 times with TPBS, the pieces were incubated with anti-PPL2A antibody. Subsequently, the pieces were washed 3 times with TPBS, and incubated with HRP-conjugated anti-mouse IgG. The detection was performed by SEM using enzymatic substrate of peroxidase, DAB in the presence of H_2_O_2_, and osmium tetroxide, which enhanced the electron density of DAB precipitate.

Immunostaining of late trochophore and D-shape larvae of *P. penguin* was performed using anti-PPL2 mouse-antibody and Alexa488 fluor-labeled anti-mouse IgG or each anti-PPL2 subunit rabbit-antibody against PPL2α, PPL2β, and PPL2γ and Alexa488 fluor-labeled anti-rabbit IgG antibody, respectively. The nuclear DNA was stained by DPAI (4′, 6-diamidino-2-phenylindole) (Invitrogen, CA). The samples were observed by using an Olympus FV1000 confocal laser-scanning microscope (Olympus, Tokyo, Japan) or fluorescence microscope BZ-X700 (Keyence Co., Osaka, Japan).

### Morpholino design and morphological phenotype analysis of morphants of *P. penguin* D-shaped larva

The knockdown experiments using morpholino oligos were conducted by reference to the previous papers for invertebrate embryos [16], [37], [38]. Translational-blocking morpholino oligos (MOs) of *P. penguin* lectin, MO-PPL2A corresponding to the antisense nucleotides from the initiator codons of PPL2A were designed to block their translation, respectively, and were synthesized using Gene Tools (Philomathe, OR, USA). MO-PPL1 was designed for PPL1 as control. These sequences are as follows: 5′-AAATAATAGACAAGGCCGATTTCAT-3′ for MO-PPL2A (γ subunit), 5′-TCAGTCCTCCAAAGGTCTTCGACAT-3′ for MO-PPL2B, and 5′-ACATAAGCACAGCTATCACCAACAT-3′ for MO-PPL1. Standard control morpholino oligo, 5′-CCTCTTACCTCAGTTACAATTTATA-3′, was from Gene Tools.

After fertilization of *P. penguin* eggs, floating blastulae were selected by decantation to synchronize the embryonic development. MO-PPL2A, MO-PPL2B, MO-PPL1 and control oligo were dissolved in nuclease-free water at 0.2 mM, and were transfected into synchronized *P. penguin* larvae at a final concentration of 10 µM for each oligo per larvae (5×10^3^ population/ml sterilized artificial sea water) at late trochophore larval stage by Endo-porter (8 µM). After incubation at 20°C overnight, the morphants and control D-shaped larvae (treated with or without Endo-porter only) were fixed by adding 20 µl of paraformaldehyde (final 2%) for SEM examination. For Western blot and LC/MS/MS analyses, each morpahnt sample and control larvae were harvested without fixiation, in part, and stored at −80°C until use. SEM images of D-shaped larvae were acquired using a Hitachi S-4200 at an acceleration voltage of 5 kV. Phenotypes of morphants (ca. 50 to 100) were counted and scored according to the relative percentage of 4 morphological types (D-shaped, small D-shaped, incomplete shell, and no shell larvae). Each experiment was conducted in duplicate and analyzed using chi-square tests.

Specific reductions of target proteins by morpholino oligos were estimated by Western blotting analysis using anti-PPL2A antibody and multiplexed relative protein quantitation by LC/MS/MS analysis combined with iTRAQ regents. Briefly, parallel multiplex labeling reactions were performed in MO-PPL2A- and MO-PPL2B treated samples and control labeled with the 117, 115 and 116 iTRAQ reporter tags, respectively. The iTRAQ labeled peptide pools were then mixed together in a equal ratio and stored at −20°C, followed by nanoLC-MS/MS analysis. The protein samples were extracted from *P. penguin* larva (∼1.0×10^3^ populations) using extraction buffer (20 mM HEPES (pH7.9), 400 mM NaCl, 1 mM EGTA, 1 mM EDTA, 1 mM DTT and 1 mM PMSF), and concentrated by acetone precipitation. After reduced by tris-(2-carboxyethyl) phosphine (TCEP), alkylated, and digested with trypsin (Wako Pure Chemical., Osaka, Japan) at 37°C overnight, the resulting peptide pools were labeled with one of multiplex set of isobaric tagging iTRAQ reagents, respectively. Then, a parallel set of reactions was combined, desalted by using Sep-Pak cartridge column (Waters Associates, Milford, MA), subsequently separated by using a DiNa Nano LC system equipped with a DiNa MALDI spotting device (KYA Technologies Co., Tokyo, Japan). The peptides were eluted from a reversed phase-HiQ sill C18 column by using a binary gradient of acetonitrile in 0.1% trifluoroacetic acid. The column effluent was mixed directly with the MALDI matrix solution (4 mg/ml of CHCA and 80 µg/ml of ammonium citrate in 70% acetonitrile containing 0.1% trifluoroacetic acid) at a flow rate of 2.5 µl/min before spotting onto MALDI target plates (Opti-TOF 384-well insert; AB Sciex, Foster City, CA). The molecular mass and sequence of peptides on MALDI target plates were analyzed by using an AB SCIEX TOF/TOF 5800 Analyzer and 4000 Series Explorer software (version 4.0) (AB Sciex). Molecular masses were calibrated using the Sequazyme Peptide Mass Standards Kit or BSA TestStandard Kit (Applied Biosystems). Protein identification was performed with Protein Pilot software (version 3.0; AB Sciex) using the Paragon method. Each MS/MS spectrum was searched against a protein sequence database, NCBInr.

### *In vitro* crystallization

The *in vitro* crystallization experiments were carried out in 96-well titer plates (Non-binding surface, #3881, Corning Inc., NY, USA) by adding PPL2A or PPL2B to the artificial sea water (0.5 M NaCl and 0.011 M KCl) including 10 mM CaCl_2_ and 8 mM NaHCO_3_. The plate was kept for 48 h at room temperature, and the morphological effects of proteins on crystallization of CaCO_3_ were monitored by an optical microscope. The morphology, size and number of crystals formed in the presence of proteins were compared to those of crystals grown in parallel without protein. BSA and PPL1 were used as control. The effects of trehalose and glucose, to which specific for PPL2A, on crystallization of CaCO_3_ were also assessed with or without PPL2A. Counting the number of crystal images were conducted by MetaMorph software (Molecular Devices, LLC, CA, US). Furthermore, the localization of PPL2A and PPL2B on CaCO_3_ crystals was analyzed by using Alexa Fluor 488 (AF488)-conjugated PPL2A and AF568-conjugated PPL2B, respectively. Labelings of PPL2A and PPL2B (500 µg each) were conducted by using Alexa Fluor 488 carboxylic acid 2,4,5,6-Tetrafluorophenyl (TFP) ester or Alexa Fluor 568 TFP ester, which are more stable and resistant to nonspecific hydrolysis than succimidyl ester, according to the manufacturer’s protocols (Life Technologies Co., Carlsbad, CA USA). After purified by gel filtration and concentrated by ultrafiltration, AF488-conjugated PPL2A and AF568-conjugated PPL2B were used for *in vitro* crystallization experiment. Localization of PPL2A and PPL2B on crystals was imaged via fluorescence microscopy (Olympus IX71 equipped with Hamamatsu Digital camera C10600 ORCA-R2) using MetaMorph NX software. A mercury lamp with U-MNIBA3 filter (Ex 470–495 nm, Em 510–550 nm, dichroic filter 505 nm) and U-MWIG3 filter (Ex 530–550 nm, Em 575 nm, dichroic filter 570 nm) were used for fluorescence imaging.

The crystal system of CaCO_3_ such as calcite polycrystals was confirmed by using a laser micro-Raman spectroscopy NRS 5100 model (JASCO Co., Tokyo, Japan).

### Circular Dichroism (CD) Spectroscopy

CD spectra were measured at room temperature using a Jasco J-720 spectropolarimeter (Jasco, Tokyo, Japan) in the range of 210–260 nm with a 0.5 cm path length. Protein solutions were prepared at 0.1% (W/V) in 50 mM Tris-HCl (pH 7.5) and left for 2 h at room temperature before measurements.

## Results

### Isolation and characterization of PPL2s

**Figure 1:**
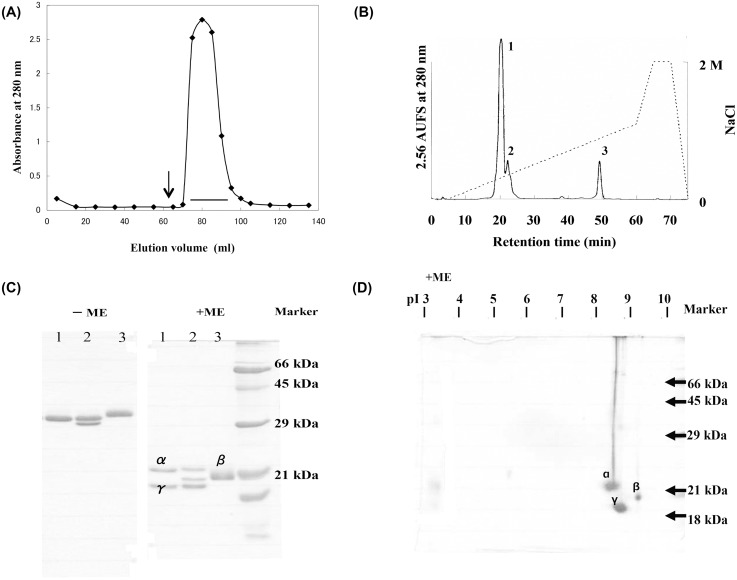
Purification of PPL2s. A, Affinity chromatography of the secreted fluid of *Pteria penguin* mantle on a trehalose-Sepharose 4B column. Arrows indicates elution buffer: 0.2 M trehalose in 10 mM Tris-HCl buffer (pH 7.5) containing 0.5 M NaCl and 5 mM CaCl_2_. B, Cation-exchange chromatography of adsorbed fraction of trehalose-affinity chromatography of secreted fluid of *Pteria penguin* mantle on a Resource S column (6.4×30 mm). Flow rate was 1 ml/min. C, SDS-PAGE (15%) profiles of PPL2A and PPL2B under non-reducing (−ME) and reducing (+ME) conditions after ion-exchange chromatography. Lanes 1, 2 and 3 correspond to fractions Nos.1, 2 and 3 in Fig. 1B, respectively. D, 2D gel electrophoresis of PPL2s.

We previously isolated a lectin named PPL1 from the mantle and the secreted fluid of *P. penguin* by affinity chromatography on mucin-sepharose as adsorbed protein [23]. Strong hemagglutinating activity was still detected in the unadsorbed fraction, and this was inhibited by trehalose, which is one of major carbohydrate components identified in the oyster, *Crassostrea virginica* [39]. Therefore, the unadsorbed fraction was further purified by affinity chromatography on trehalose-Sepharose 4B (Fig. 1A). Successive cation exchange chromatography on a Resource S column yielded three peaks (Fig. 1B). Since the first and third peaks showed strong agglutination activity toward rabbit erythrocytes, and gave a single protein band corresponding to the dimeric species on SDS-PAGE under non-reducing conditions (Fig. 1C), the lectins from the first and third peaks were designated as PPL2A and PPL2B, respectively. On the other hand, second peak contained two protein bands corresponding to PPL2A and other low-molecular-weight protein designated as PPL2C on SDS-PAGE under non-reducing conditions (Fig. 1C). The 2D-PAGE profile for PPL2 including PPL2A, 2B and 2C showed only three distinct spots corresponding to α, β, and γ subunits, of which isoelectric points were estimated to be 8.5, 9.3, and 8.8, respectively (Fig. 1D). Furthermore, PPL2A gave two bands on SDS-PAGE in the presence of 2-mercaptoethanol, while PPL2B gave a single band (Fig. 1C). This indicated that PPL2A and PPL2B existed as a hetero-dimer of α and γ subunits and a homo-dimer of β subunit cross-linked by intermolecular disulfide bonds, respectively. While, second peak containing PPL2A and PPL2C gave three bands corresponding α, β and γ subunits under reducing condition (Fig. 1C, lane 2). Judging from the results of SDS-PAGE and 2D-PAGE analyses (Figs. 1C and 1D), that is, PPL2A is composed of α and γ subunits, and the protein band corresponding to the γ subunit was more densely stained (Fig. 1C, lane 2), PPL2C seems to be hetero-dimer of β and γ subunits. Due to the very low-yielding of PPL2C and the common components of PPL2C with PPL2A and PPL2B, PPL2C has not been further investigated.

To investigate the sugar-binding specificities of PPL2A and PPL2B, a number of carbohydrates including trehalose (α1, 1) and its related sugars containing α-glucosidic linkage such as kojibiose (α1, 2) and isomaltose (α1, 4) were used. In the hemagglutination inhibition assay using rabbit erythrocytes, isomaltose and trehalose were the most potent saccharide inhibitors for PPL2A (Table S2 in File S1). D-GlcNAc and D-fructose showed inhibitory effects toward PPL2A. On the other hand, kojibiose showed only weak inhibitory effect for PPL2B among mono or oligo saccharides. Asialofetuin showed strong inhibitory activities against both PPL2A and PPL2B, while asialomucin (types I and II) and heparin showed no inhibitory activity. Sialoglycoproteins such as fetuin and mucin showed inhibition against PPL2B but not against PPL2A. Thus, PPL2A and PPL2B had clearly different carbohydrate-binding specificities.

### Identification of free sugars in *Pteria penguin* organs

PPL2A showed unique carbohydrate binding activity for trehalose as mentioned above. Thus, to identify the presence of sugars and polyols in *P. penguin* organs, the free sugars extracted from *P. penguin* organs including adductor muscle, gill, body fluid and mantle, were analyzed by TLC. Single sugar was mainly detected in the body fluid, while some carbohydrates were detected in the adductor muscle, gill and mantle (Figure S1 in File S1). The main sugar in the body fluid gave the same R_f_ value as sucrose, which was converted into glucose and fructose by treating with invertase, and gave glucose spot (Figure S1 in File S1). On the other hand, glucose, maltose and maltotriose were detected in the adductor muscle, while trehalose and sucrose were detected in mantle (Figure S1 in File S1). Thus, trehalose, which is one of specific binding sugars of PPL2A, was found to be abundant in *P. penguin* mantle.

### Amino acid sequences of PPL2 subunits

**Figure 2:**
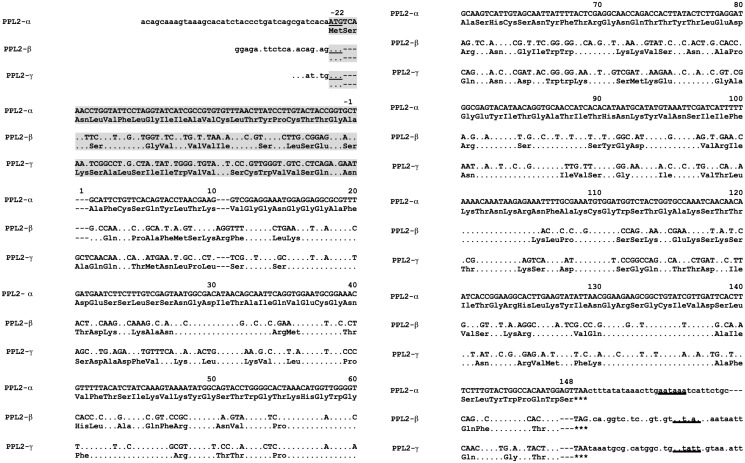
Nucleotide sequences of cDNAs and the corresponding amino acid sequences of PPL2 subunits. Nucleotide residues and amino acid residues identical to those of PPL2α subunit are indicated by ‘dot’ symbol, respectively. Dash (–) symbol indicates the deletion of nucleotides and amino acids. Underline indicates the putative polyadenylation signal, AATAAA. Shaded region indicates the signal peptide region.

**Figure 3:**
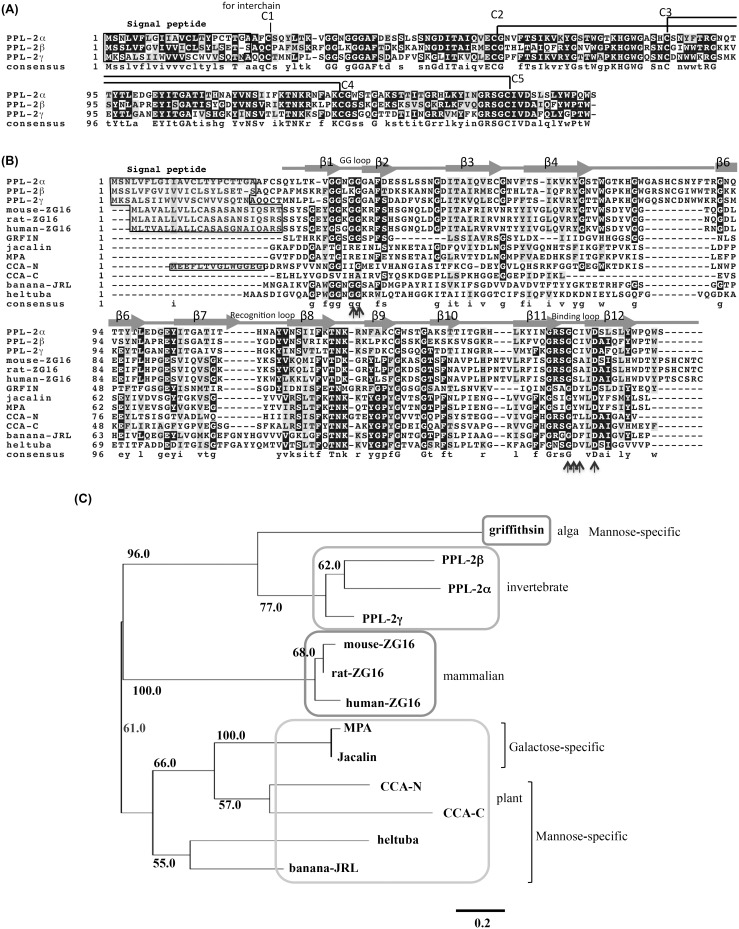
Aligned amino acid sequences of PPL2 subunits (A), jacalin-related lectins (B), and phylogenetic tree of the CRDs of jacalin-related lectins (C). The sequences were aligned using Clustal W program and represented by using BOXSHADE 3.21. The residues identical to the column-consensus were presented by inverse character (black background), while the residues, which are not identical but at least similar to the column-consensus, were presented by gray background. C1 to C5 indicates the half cysteine residues with inter- and intra-disulfide bonds. Secondary structural element (β strands) were shown as arrows (β1–β11). The GG loop, recognition loop, and binding loop were parts of carbohydrate recognition domain. Phylogenetic tree was constructed by the improved neighbor-joining algorithm, BIONJ, using the PhyML (ver 3.1) program with the aligned sequences treated with Gblocks program in SeaView package (ver. 4.5.3). The numbers associated with the nodes are the percent reproductions of branches in 1000 bootstrap reconstructions. Scale bar: 0.1 substitutions per site. Jacalin: Galactose-binding lectin from *A. integrifoli*a; MPA: *M. pomifera* lectin; BanLec: *M. acuminata* lectin; CCA: *C. crenata* lectin.

After PPL2A and PPL2B were reduced and *S*-carboxamidomethylated, respectively, their subunits (α and γ subunits for PPL2A, β subunit for PPL2B) were separated by reversed-phase HPLC and subjected to N-terminal sequencing. The full-length amino acid sequences of PPL2A (α and γ subunits) and PPL2B (β subunits) were determined by direct amino acid sequencing using intact and several peptide maps generated by *Achromobacter* protease I, V8 protease, and *Clostridium histolyticum* clostripain digestions (Figure S2 and Table S3 in File S1) and cDNA sequence analysis using RACE method, respectively. Thus, the cDNA encoding the PPL2α subunit included 577 nucleotides with an open reading frame of 504 nucleotides encoding for a mature protein of 146 amino acid residues, and a signal peptide of 22 residues (Fig. 2). Meanwhile, both subunits of PPL2β and PPL2γ were composed of an open reading frame of 501 nucleotides encoding for a mature protein of 146 amino acid residues and a signal sequence of 21 residues (Fig. 2). Thus, all of the α, β, and γ subunits were composed of 146 amino acid residues with approximately 50% sequence identity among them (Fig. 3A). Although the calculated molecular masses of β and γ subunits (16551.18 and 16199.51 Da, respectively) were in agreement with the experimental values, 16512.57 Da and 16116.25 Da, from MALDI-TOF–MS, respectively, the molecular mass of α subunit estimated to be 15934.71 Da from the primary sequence was not consistent with the observed ones, 17211.12 and 17358.34 Da, from MALDI-TOF-MS (data not shown). This discrepancy was assumed to be post-translational modification, because the PPL2α subunit had an Asn70-X-Thr72 consensus motif for *N*-glycosylation, and two types of *N*-linked sugar chains were detected in the PPL2 αsubunit (Figure S3 and Table S4 in File S1). Furthermore, the position of the disulfide bond pairing in PPL2A and PPL2B was analyzed by peptide mapping with endoproteinase Lys-C digestion (Figure S4 in File S1). PPL2B, which was composed of two identical βsubunits, gave two fragments, of which molecular masses were measured to be m/z 3914.80 (calculated as 3913.44) and m/z 4298.03 (calculated as 4288.85). This yielded two amino acid sequences corresponding to Ala25–Lys55 and Cys109–Lys113 of β subunit and His56–Lys71 and Tyr126–Trp146 of the other β subunit, respectively. This indicates that two intrachain disulfide bonds, Cys37-Cys109 and Cys63-Cys134, were present in PPL2B (Figures S4 and S5 in File S1). On the other hand, PPL2A composed of two polypeptides, α and γ subunits, gave fragments for the assignment of two intrachain disulfide bonds, Cys37-Cys109 and Cys63-Cys134 of γ subunit (Figures S4 and S5 in File S1). Furthermore, Cys62-Cys133 of α subunit was detected by sequencing, and other disulfide bonds pairing were estimated by its sequence homology with β and γ subunits.

BLAST research, multiple sequence alignment and phylogenetic analysis of PPL2s subunits, PPL2α, PPL2β and PPL2γ, revealed that each PPL2 subunit showed the sequence homology with the jacalin-related lectins (JRLs), and only eight amino acid residues in CRDs were invariant between JRLs (Figs. 3B and 3C).

### Preparation and characterization of anti-PPL2 antibodies

Anti-PPL2A and anti-PPL2B mouse antisera were prepared by injections of purified PPL2A and PPL2B, respectively. The specificities of these antibodies were checked by dot blot and Western blot analyses, showing that anti-PPL2A antibody cross-reacted with PPL2B βsubunit in addition to PPL2A α and γ subunits (Figure S6 in File S1). On the other hand, anti-PPL2B mouse antibody with sufficient reactivity could not be obtained.

To obtain more specific antibodies against each subunit, PPL2α, PPL2β, and PPL2γ, polyclonal antibodies were prepared by immunization of rabbits with the synthetic peptide epitope-KLH conjugates, respectively. The specificities of antibodies were checked by Western blot analyses (Figure S6C in File S1). They can recognize specifically each subunit of PPL2s without any cross-reactivity among PPL2s subunits, respectively.

### Immunohistchemical analysis of PPL2A and PPL2B in lavae cells

**Figure 4:**
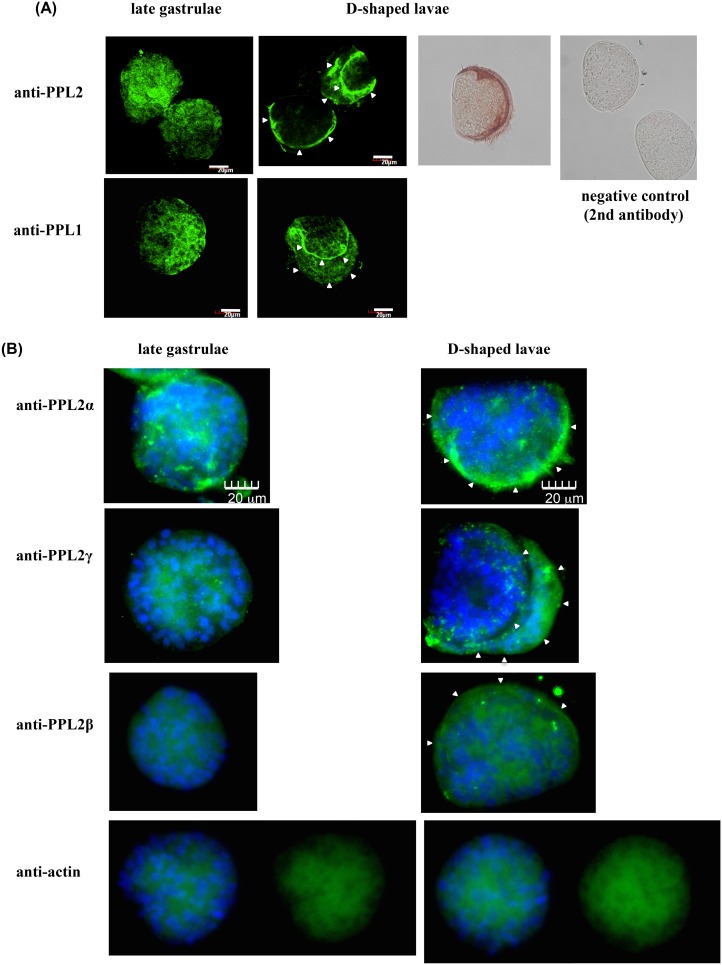
Distribution of *Pteria penguin* lectins, PPL2 s, in the late gastrulae and D-shaped lavae. A, Immunostaining of late trochophore and D-shape larva of *Pteria penguin* was performed using anti-PPL1 and anti-PPL2A mouse antibodies, combined with AlexaFluor488-labeled anti-mouse IgG antibody or HRP-conjugated anti-mouse IgG antibody. Arrowheads indicate the mantle. B, Immunostaining of late trochophore and D-shape larva of *Pteria penguin* using specific antibodies against PPL2 α, β and γ subunits. Anti-actin antibody was used as a control. Arrowheads indicate the mantle.

Figures 4A and 4B shows the immunohistochemical analysis of PPL2 subunits and PPL1 at the late gastrula and D-type larva stages. These demonstrat that the PPL2 subunits, especially PPL2αand PPL2γ subunits, in addition to PPL1 distributed in most cells of late gastrula and in the mantle of D-type shells.

### Knockdown analysis of PPL2A by using morphants of Pteria penguin D-shaped larva

**Figure 5:**
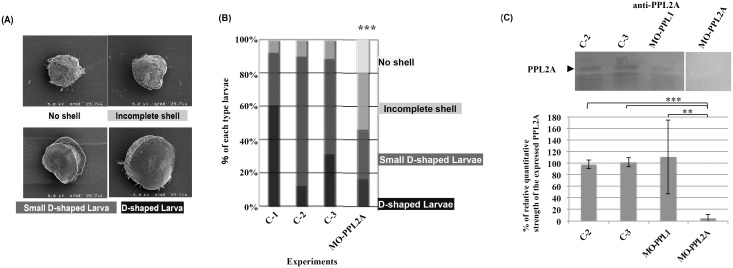
Knockdown analysis of PPL2A on D-shape shell formation at larval stage. A, Typical SEM images of D-shaped larva with or without morpholino oligos. Larva phenotypes could be divided into four groups, no shell, incomplete shell, small D-shaped larva, and D-shaped larva. B, Frequency of the phenotypes obtained by knockdown analysis of PPL2A. Controls C-1: treated without Endo-porter, C-2: treated with Endo-porter, C-3: treated with Endo-porter and control oligos, MO-PPL2A: treated with morpholino oligo for PPL2A. Each experiment was conducted in duplicate and analyzed using chi-square tests (df = 2). ***: p<0.001. C, Western blotting analysis of morphants with specific reduction of targeted protein by using anti-PPL2A antibody after separated by SDS-PAGE with non-reducing condition. The detection was carried out with chemiluminescence method using ECL Plus regent. Relative quantitative strength of PPL2A was estimated from the independent chemiluminescence images (n = 3) acquired by a chemiluminescent imaging system equipped with highly-cooled CCD cameras, AE9300-Ez-Capture MG, and analyzed by densitograph software. The data were evaluated using t-test. ***: P<0.001, **: P = 0.098.

To analyze the direct involvement of *P. penguin* lectins in the biomineralization process, knockdown studies were conducted for D-shell structure formation at larval stage using translational-blocking MOs specific to PPL2A (γ subunit). After introducing MOs of each gene, the rate of D-shell formation for each experiment was estimated by scoring 4 morphological types determined from SEM micrographs (Figs. 5A and 5B, and Figure S7 in File S1).

Specific reduction of targeted protein was assessed by Western blotting analysis using anti-PPL2A antibody and the multiplexed relative protein quantitation by nanoLC-MALDI-TOF/TOF-MS analysis combined with iTRAQ regents, respectively (Fig. 5C and Table S5 in File S1). Compared with the controls, Endo-porter treated or control oligo-soaking, PPL2A expression levels decreased to <10% with MO-PPL2A soaking group (Fig. 5C). To determine the relative ratios of expressed proteins in D-shell larvae, multiplexed relative quantitation was also conducted by iTRAQ-LC-MALDI-TOF/TOF-MS analysis. It showed the specific reduction of PPL2A for MO-PPL2A-treated sample (decreased to 34–48%) compared with control (untreated group) although the detected peptide fragments were very limited to small part of proteins including PPL2A in the nanoLC-MALDI-TOF/TOF-MS analysis (Table S5 in File S1).

The percentage of complete D-shaped larva including small size was similar among three control experiments (84%–92%). These results indicated that there was no effect of Endo-porter and/or control MOs on the morphology during *P. penguin* larva development although some effects of Endo-porter were detected on the size of D-shaped larva (Fig. 5B). In contrast, 54% of larva treated with MO-PPL2A had no shell (20%) and incomplete shell (34%); only 16% had normal D-shaped shell formation (Fig. 5B). On the other hand, PPL1 showed no effect on *P. penguin* larva development (Figure S7 in File S1). Thus, the knockdown of PPL2A expression affected D-shaped shell formation, suggesting that PPL2A γ subunit affected on the shell molphologies *via* two possibilities as a regulatory factor causing the disruption of a number of different developmental processes and/or as a shell matrix construction material.

### Detection of PPL2 subunits in the nacreous layer of shell as matrix proteins

**Figure 6:**
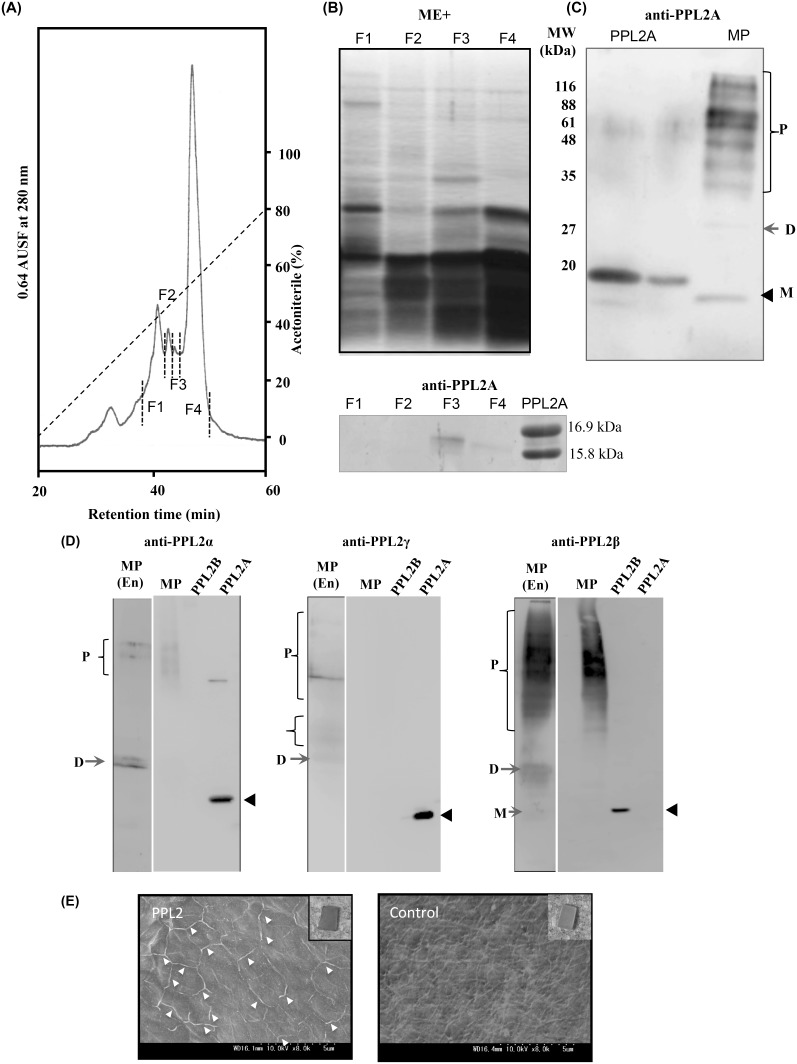
Identification of PPL2 in the *P. penguin* nacreous layer. A, Purification of matrix proteins from *P. penguin* nacreous layer by RP-HPLC. B, SDS-PAGE profiles and Western blot analysis of fractions of nacreous layer matrix proteins (F1 to F4) from *P. penguin* under reducing conditions. Proteins were detected by silver staining. C, Western blot analysis of nacreous layer matrix proteins and PPL2A using anti-PPL2A antibody. D, Western blot analysis of nacreous layer matrix proteins (MP), PPL2A and PPL2B using specific antibodies against PPL2 α, β, and γ subunits, respectively. Chemiluminescence signals were detected by CCD cameras with exposure time of 2∼3 sec. MP(En) indicated the experiments with enhanced exposure time of 30 sec. M: monomer, D: dimer, P: polymer. E, *In situ* localization of PPL2 as an organic matrix protein in the nacreous layer detected by combined anti-PPL2A antibody (mouse) and HRP-conjugated anti-mouse IgG 2ndary antibody. HRP was detected by DAB stain and enhanced by osmium tetroxide treatment. Arrowheads indicate the positive staining by anti-PPL2A antibody with HRP-conjugated anti-mouse IgG antibody and DAB-OsO_4_. Control was treated with HRP-conjugated anti-mouse IgG antibody and DAB-OsO_4_ staining without anti-PPL2A antibody.

To directly identify whether these lectins exist in the matrix proteins from *P. penguin* nacreous layer, we extracted the matrix proteins from the nacreous layer by 70% formic acid after decalcification with 0.5 M EDTA, and partially purified by RP-HPLC (Fig. 6A), and analyzed by Western blotting with anti-PPL2A antibody (Figs. 6B and 6C). Western blot analysis of fractions (F1 to F4) partially purified by RP-HPLC showed that the 16 kDa band corresponding to the β or γ subunit of PPL2 was detected by anti-PPL2A antibody (Figs. 6A and 6B). However, this anti-PPL2A antibody-positive band could not be distinguishable from the subunits of PPL2 because anti-PPL2A antibody showed the cross reactivity against PPL2B (Figure S6 in File S1). Furthermore, several positive bands against anti-PPL2A antibody were detected at the high molecular mass regions ranging from 30 to 120 kDa (Fig. 6C), raising the possibilities the existence of polymerized forms of PPL2s in nacreous layer or non-specific immuno-reactivity. Thus, to study this possibility and address these problems by identifying each subunit of PPL2s in the nacreous layer matrix proteins, we conducted Western blot analysis using newly prepared PPL2 subunit-specific antibodies, anti-PPL2α (3–17), anti-PPL2β (14–30), and anti-PPL2γ (64–79) rabbit polyclonal antibodies. These analyses show that PPL2 subunits exist mainly as a dimer and higher molecular mass crosslinked polymeric forms (Fig. 6D), and only PPL2B (β subunit) was detected as a monomer in part (Fig. 6D) although they existed mainly as a monomer (under reducing condition) in the secretory fluid of mantle (Figure S6D in File S1). Furthermore, using immunohistochemistry of PPL2A, PPL2 matrix proteins were localized at the boundary regions of adjacent aragonite crystal micrograins or the emergence region of the polycrystalline on the nacreous layer (Fig. 6E). These results suggest that the PPL2 subunits contribute biomineralization mechanism in the nacreous layer of *P. penguin* as matrix proteins. Preliminary 2D PAGE analysis of nacreous layer matrix proteins showed that some acidic spots were shifted to basic ones corresponding to the PPL2s by bacterial alkaline phosphatase (BAP) treatment (Figure S8 in File S1). These results suggest that PPL2s might be phosphorylated and exist as acidic matrix proteins in nacre.

### *In vitro* crystallization

**Figure 7:**
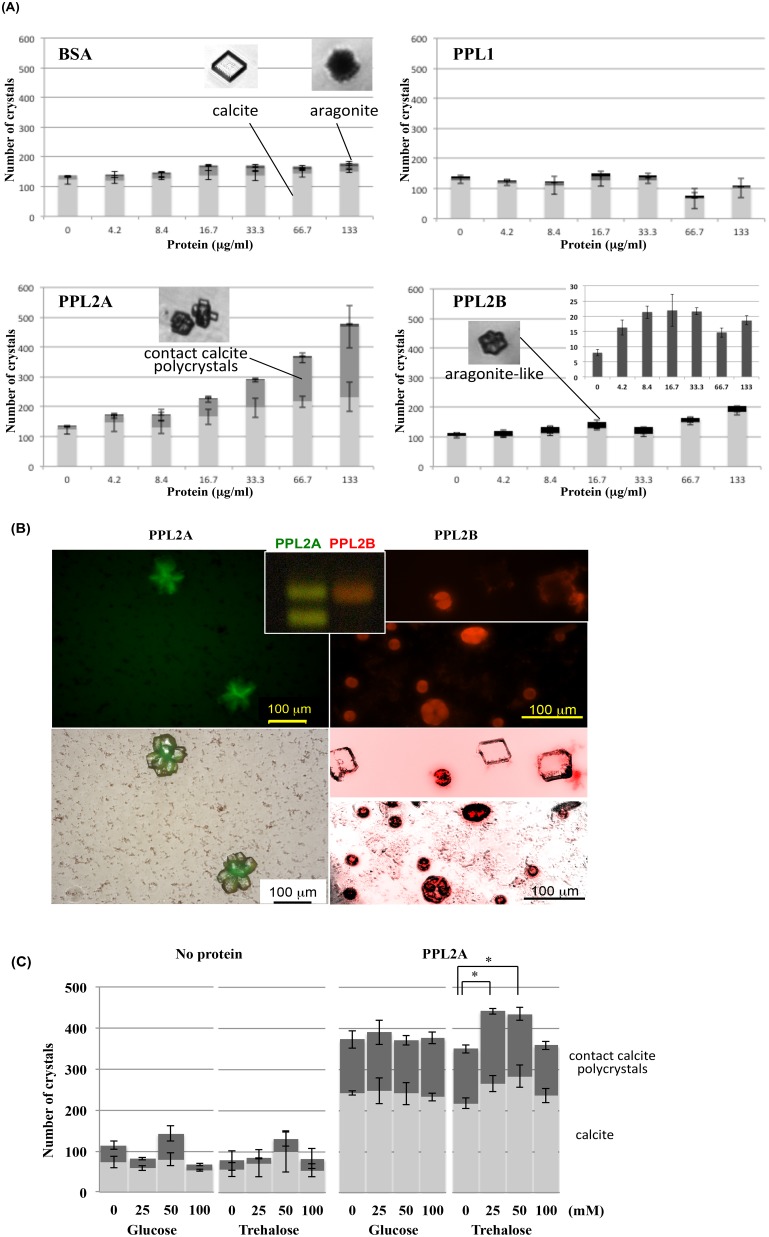
Effects of PPL2A and PPL2B on the *in vitro* CaCO_3_ crystallization. A, Effects of PPL2A and PPL2B on the number and morphology of CaCO_3_ crystals. BSA and PPL1 were used as controls. *Insert*: the effect of PPL2B on the number of aragonite crystals at different concentrations. B, Fluorescence microscopy imagings of Alexia488-labeled PPL2A (left) and Alexia568-labeled PPL2B (right) on CaCO_3_ crystallization (upper). The superimposed optical images collected by using bright-field and fluorescence microscopy (lower). C, Effects of trehalose and glucose on the CaCO_3_ crystallization with/without PPL2A. *Insert*: SDS-PAGE profiles of fluorescence-labeled PPL2A (green) and PPL2B (red).

To examine whether PPL2A and PPL2B affect on the calcium carbonate crystallization directly, *in vitro* crystal growth experiments were performed by using PPL2A and PPL2B in addition to PPL1 and BSA. Optical microscope images showed that the number of contact polycrystalline calcites, of which morphology was quite different from a calcite crystal, was increased depending on the concentration of PPL2A, while only the calcite crystal having a trigonal system were formed without PPL2A. This polycrystalline calcite was confirmed to be calcite by microscopic Raman spectroscopy (Figure S9 in File S1). While BSA or PPL1 showed no effect on the morphology and number of crystals (Fig. 7A). Thus, the rate of polycrystalline CaCO_3_ tended to be increased according to the concentration of PPL2A (Fig. 7A). On the other hand, PPL2B showed the small but distinct dose-dependent increasing of the number of floriform (flower petal-like shape) crystals at the low concentration ranging from 0 to 16.7 µg/ml (Fig. 7A).

In order to further elucidate how PPL2A and PPL2B act on crystal formation, the distributions of PPL2A and PPL2B in crystals were investigated by using AF488 fluorescence-labeled PPL2A and AF568-labeled PPL2B, respectively (Fig. 7B). On the crystallization condition with AF488-labeled PPL2A, it located at the boundary regions of contact crystals or grain boundary of the polycrystalline (Fig. 7B). These observations suggest that PPL2A showed the critical role for calcium carbonate crystallization as a matrix protein. These results also agree with the immunohistochemical observation showing the specific localization of PPL2 at the boundary regions of crystals (Fig. 6E). While, AF568-labeled PPL2B located on the aragonite and floriform crystals (Fig. 7B), indicating that the PPL2B is likely to facilitate the aragonite crystal formation. Thus, these results indicate that PPL2A and PPL2B have different roles on the CaCO_3_ crystallization.

Furthermore, the effects of trehalose and glucose on the CaCO_3_ crystallization by PPL2A were assessed (Fig. 7C). Interestingly, trehalose had increasing effect of crystal formation with PPL2A, but not without PPL2A, indicating that the trehalose affected the biomineralization functions of PPL2A.

### Circular Dichroism (CD) Spectroscopy

To examine the secondary structures of PPL2A and PPL2B, far-UV circular dichroism (CD) spectrum was measured. From the deconvolution of CD spectra (Figure S10 in File S1), both PPL2A and PPL2B showed low α-helix contents (∼5%) and high β-sheet contents (∼50%), and random coil (∼45%). Therefore, PPL2A and PPL2B were similar secondary structure mainly composed of β-sheet structure same as other JRLs.

## Discussions

Previously, we isolated PPL1 from the mantle and secreted fluid of *P. penguin* [23]. PPL1 was a monomeric protein of 18.0 kDa, which includes two tandem-repeat domains being homologous to the RBLs from fish eggs. PPL1 has strong agglutinating activity against Gram-negative bacteria including *Aeromonas salmonicidae* [23]. Here, we isolated and characterized two novel jacalin-related lectins (JRLs), PPL2A and PPL2B, from the mantle of *P. penguin*.

PPL2A was composed of covalently linked hetero-dimers of 16.5 and 15.8 kDa (α and γ) subunits, and PPL2B was composed of covalently linked homo-dimer of 16.0 kDa (β) subunit, respectively, as shown in Fig. 1C. The PPL2 subunits, PPL2α, PPL2β and PPL2γ, had extensive sequence homology to JRLs, which are also known as β-prism fold lectins, from the tubers of the Jerusalem artichoke (*Helianthus tuberosus*; Heltuba) of *Asteraceae* [40], leaves from salt-stressed rice of *Gramineae* [41], seeds from *Pakia platycephala* [42], pulp of banana (*Musa acuminata*; BanLec) fruits of *Musaceae* [43] and the seeds of the Japanese chestnut (*Castanea crenata*; CCA) of *Fagaceae* [44] (Fig. 3B). This indicates that PPL2s are novel JRLs and the first to be isolated from invertebrates. Multiple sequence alignment of PPL2s subunits with JRLs revealed that only eight amino acid residues were invariant between JRLs (Fig. 3). These residues are important for establishing the common structural feature including the GG loop and binding loop (Gly132, Asp136) at the top of the β-prism fold, which function as part of carbohydrate recognition domain (CRD) of JRLs (Fig. 3B) [45]–[47]. While, the recognition loop at CRD showed the variation in length and sequences. These results suggest that PPL2A and PPL2B possess one CRD per subunit, and are active as a dimer as well as other JRLs.

JRLs have been classified into two groups, a galactose-specific group (gJRLs) and a mannose-specific group (mJRLs), according to their sugar specificities (Fig. 3C). gJRLs including jacalin and Maclura pomifera lectin (MPA) are composed of cleaved protomers consisting of a β-chain (20 amino acids) and an α -chain (133 amino acids). Proteolytic processing of the pro-proteins generates a new N-terminal glycine residue, which forms hydrogen bond with O-4 of galactose, to shift its sugar-binding specificity toward galactose residues in jacalin and MPA [48]. Therefore, the carbohydrate-binding specificities of PPL2s might differ from those of jacalin and MPA because of the lack of proteolytic cleavage in their polypeptide chains. Practically, PPL2A and PPL2B showed no affinity to glucose and mannose that are different carbohydrate specificities from those of gJRLs and mJRLs. Furthermore, PPL2A and PPL2B had very different sugar-binding specificities each other, that is, PPL2A recognized isomaltose and trehalose while PPL2B weakly recognized only kojibiose, although they showed the sequence similarities (Table S2 in File S1). In the purification step of PPL2A and PPL2B by affinity chromatography on trehalose-Sepharose 4B, PPL2B was detected in the adsorbed fraction accompany with PPL2A in spite of no inhibition of PPL2B by trehalose. This discrepancy can be explained by that PPL2B interacts with PPL2A *via N*-linked sugar chains during purification.

A phylogenetic tree based on the amino acid sequences of 13 JRLs including PPL2s (Fig. 3C) revealed that the clusters of JRLs were divided into four groups: pancreatic 16 kDa protein ZG16p from mammalian [46], [49], griffithsin from alga [50], plant JRLs, and PPL2s from invertebrate.

In this study, to assess the involvement of *P. penguin* lectins in the biomineralization process, knockdown studies of PPL2A was conducted for D-shell structure formation at larval stage using molpholino oligos. The knockdown by MO-PPL2A showed distinct effects on D-shell structure formation but not PPL1 (Fig. 5 and Figure S7 in File S1), suggesting that PPL2A was involved in the shell formation during the late larva to D-type larva stage (Fig. 5). Preliminary knockdown experiment of PPL2B also showed the defective development effects on D-shell structure formation at larval stage as well as PPL2A although the PPL2B expression in morphant could not be sufficiently assessed by Western blot and iTRAQ method probably due to the low-expression yields of PPL2B in larvae and/or post-translational modifications (Figure S7 in File S1). Moreover, we observed that PPL2s were distributed and included in the nacreous layer of *P. penguin* as matrix proteins (Fig. 6). PPL2s were primarily localized in the boundary surfaces between the aragonite crystals (Fig. 6E).

*In vitro* CaCO_3_ crystallization analysis also showed that PPL2A affect directly on the crystallization of calcite, that is, PPL2A increased the number of contact polycrystalline calcite by binding to the boundary regions of crystals (Fig. 7). This *in vitro* observation agreed with the result in nature showing the specific localization of PPL2A at the boundary region of aragonite crystals or the emergence region of the polycrystalline on nacreous layer (Fig. 6E). On the other hand, PPL2B induced the formation of floriform (flower petal-like shape) crystals by incorporating into the crystals (Fig. 7). Thus, it was suggested that PPL2A and PPL2B but not PPL1 play major but different individual roles in the biomineralization of *P. penguin* nacre. Furthermore, it was found that trehalose, which was one of specific sugars recognized by PPL2A (Table S2 in File S1) and abundant in *P. penguin* mantle (Figure S1 in File S1), had increasing effect of crystal formation with PPL2A (Fig. 7C). These results suggest that the biomineralization functions of PPL2A might be regulated by trehalose in mantle *via* specific binding. It has been reported that trehalose is found in bacteria, yeast, fungi, plants, and invertebrates, but not in mammalian cells [51], [52], to serve as a carbon source, storage carbohydrate, and stress protectant. Trehalose can also function as a compatible solute to stabilize cells during osmotic stress, and its accumulation has been widely implicated in preserving cell viability during exposure to a range of environmental stresses, including heat shock, dehydration, and hypoxia [52]–[54]. In the case of bivalve, trehalose was identified in the oyster, *Crassostrea virginica Gmelin*, and found to be present throughout the period of one year in the range from 0.188 mg/g wet wt. to 3.300 mg/g wet wt [39]. Our data indicates a possible novel function of trehalose on biomineralization of shell.

The important common feature of biomineralization proteins is considered to be acidic component containing aspartate and phosphoserine residues [1], [55], [56], β-sheet structure [20], [21], and/or the presence of functional domains such as the carbonic anhydrase domains found in nacrein [57], NG repeated domain of N66 [58], a C-type lectin domain detected in the abalone nacre protein perlucin [59], and whey acidic protein (WAP) domains [60] of perlwapin [61]. These domains may be involved in protein function during the biomineralization process. In particular, β-sheet structure is advantageous for the self-assembly of insoluble matrix proteins since highly ordered β-sheet fibrils are known to self-assemble into monolayers at interfaces [54], [62] and promote the assembly of amyloid proteins such as Alzheimer’s amyloidosis [63]. Our results suggest that PPL2s contribute to the biomineralization of nacreous shells as matrix proteins although they are basic proteins in the mantle and the secreted fluid of *P. penguin* (Fig. 1D). From the sequence homology with jacalin-related β-prism fold lectins and the CD spectra (Figure S10 in File S1), it was found that both PPL2A and PPL2B adopted the β-sheet rich conformation. Furthermore, 2D-PAGE analyses of matrix proteins showed that PPL2A and 2B might be phosphorylated and exist as acidic matrix proteins in nacre (Figure S8 in File S1). Thus, it was suggested PPL2A and 2B share some critical features for biomineralization. However, more research is required to determine the mechanisms for PPL2s functions during the biomineralization process because their structural changes during biomineralization processes including polymerization and phosphorylation of PPL2 subunits are still unknown.

Several proteinous components of shell matrixes in both nacreous (aragonite) and prismic (calcite) layers have been identified in mollusc shells, and their primary structures have been determined [8]–[19], [64]. Recently, transcriptome and proteome analysis of biomineralization-related genes in the *Pinctada* species (*P. fucata*, *P. margaritifera*) were conducted [65]–[69]. Although some lectins homologous to the CTL, galectin and RBL families have been implicated in shell formation in *P. fucata* [69], matrix proteins being homologous to the JRLs were not detected in these species. This suggests that the biomineralization mechanisms and their component proteins may differ among species, even within the same family of *Pteridae* bivalve mollusks. Thus, these JRLs in *P. penguin* represent a new class of biomineralization-related genes, and underscore the widespread distribution and unique molecular evolution present within this lectin family.

### Accession Numbers

The nucleotide sequence data reported in this study were deposited in DDBJ, EMBL, and GenBank databases under accession numbers AB425237, AB425238, and AB425239.

## Supporting Information

File S1 **Supporting information. Figure S1. Silica gel TLC profiles of sugars and polyols extracted from** ***Pteria penguin*** **organs.** Reference sugars were maltotriose (Maltri), maltose (Mal), sucrose (Suc), trehalose (Tre), and glucose (Glc). Polyols and free sugars samples were isolated from the adductor muscle (Ad), gill (Gi), body fluid (Bf) and mantle (Ma) of *Pteria penguin*, respectively. +Inv indicates the invertase-reatment, which convert the sucrose to glucose and fructose. TLC was developed by ethylacetate/acetic acid/methanol/water (60∶15∶15∶10). Sugars on the plate were visualized by the orcinol-sulfuric acid method. Arrows indicate the migration of trehalose spot. **Figure S2. Reverse phase HPLC of peptides generated by proteolytic digestion of CAM-PPL2s.** CAM-PPL2s (α, β, and γ subunits) were digested with endoproteinase Arg-C (A), with *Acromobacter* protease I (B) and *S. aureus* V8(C), respectively. Peptides were separated by reversed-phase HPLC on a TSKgel ODS 120T column (5 µm, 4.6 Å∼250 mm) using a linear gradient increase of acetonitrile in 0.1% trifluoroacetic acid. The flow rate was 1 ml/min. Peptide maps for α subunit (upper), for βsubunit (middle), and for γ subunit (lower). **Figure S3. MALDI-TOF mass spectrometry of glycopeptides derived from PPL2A α subunit before (A) and after glycopeptidase A treatment (B).** Glycopeptide was digested using 0.2 mU of glycopeptidase A (Seikagaku Kogyo, Japan) and purified by reversed-phase HPLC on TSKgel ODS 120T column (4.6 mm Å∼250 mm, Tosoh), with monitoring by the phenol-sulfuric acid method. The purified sugar chains were reductively aminated with 2-aminopyridine and boranedimethylamine complex. The PA-sugar chains were analyzed using a 2D mapping method with 2 different kinds of columns; TSKgel Amide-80 column (4.6 mm Å∼250 mm, Tosoh) at a flow rate of 0.5 ml/ml at 40°C using 2 solvents, 3% acetic acid in water with triethylamine (pH 7.3) and acetonitrile (35∶65 by volume), and 3% acetic acid in water with triethylamine (pH 7.3) and acetonitrile (50∶50 by volume), and detected by fluorescence (Ex/Em = 320/380 nm). The retention time of unknown PA-sugar was converted to glucose units, which were estimated by the elution time of standard, PA-isomaltooligosaccharide mixtures. **Figure S4. Peptide mapping for determination of disulfide bonds of PPL2s.** Chromatogram of Lys-C digestion of PPL2A and PPL2B (A), and MALDI-TOF mass spectrometry of fragments including disulfide bonds derived from PPL2A (B) and 2B (C). **Figure S5. Disulfide bond structures of PPL2 subunits.** Positions of the disulfide bonds in PPL2 subunits were identified by analyzing the peptide fragments, C2(37)-C4(109) and C3(63)-C5(134) for β subunit and C2(37)-C4(109) and C3(63)-C5(134) for γ subunit, respectively, which were derived from the unmodified proteins upon endoproteinase Lys-C cleavage, and subsequent analyses by a protein sequencer and MALDI-TOF mass spectrometer. **Figure S6. Specificity of anti-PPL2A antibody checked by dot blot (A) and Western blot analyses (B), and specificities of anti-PPL2 α, β, and γ** **subunits antibodies (C) and Western blot profiles of anti-PPL2 subunits antibodies for the secretory fluid of mantle (Ma) and nacreous matrix proteins (MP) of** ***P. penguin*** **(D). Figure S7.** Knockdown analysis of PPL1 and PPL2B. Controls C-1: treated without Endo-porter, C-2: treated with Endo-porter, C-3: treated with Endo-porter and control oligos, MO-PPL1: treated with morpholino oligo for PPL1, MO-PPL2B: treated with morpholino oligo for PPL2B. Each experiment was conducted in duplicate and analyzed using chi-square tests (df = 2). ***: p<0.001. **Figure S8.** 2D PAGE analysis of matrix proteins of *P. penguin* nacreous layer with/without dephosphorylation. 2D PAGE analysis of nacreous matrix proteins were carried using the IPGphor, isoelectric focusing (IEF) system with IPG strips (3-11NL) and SDS-PAGE on 15% slab gels. +BAP: Dephosphorylation of nacreous matrix proteins by incubating with bacteria alkaline phosphatase (BAP, 0.6 U) at 30°C overnight. Some acidic spots were shifted to basic corresponding to PPL2s by BAP treatment (indicated by box). **Figure S9. Microscopic laser Raman spectroscopy of** CaCO_3_ polycrystalline derived from PPL2A. Micrographs of crystals (A) and typical Raman spectrum for calcite characterized by peaks at 1090 cm^−1^ and 279 cm^−1^ (indicated by arrows) (B). **Figure S10. CD spectra of PPL2A and PPL2B.** CD spectra were measured at room temperature using a Jasco J-720 spectropolarimeter (Jasco, Tokyo, Japan) in the range of 210–260 nm with a 0.5 cm path length. Protein solutions were prepared at 0.1% (W/V) in 50 mM Tris-HCl (pH 7.5). **Table S1. Primer sequences for 5′-, 3′- RACE and conditions of PCR amplification. Table S2. Inhibition of hemagglutination activity of PPL2s by saccharides. Table S3. Amino acid sequences of peptide fragments derived from PPL2A (α, γ) and 2B (β).** Peptide fragments designated L, R, and V were corresponding to the digests with *Acromobacter* protease I, endoproteinase Arg-C and *S. aureus* V8, respectively. Mass numbers (observed) of peptides were determined by MALDI-TOF MS analysis and compared with those calculated from the sequences. **Table S4. Structures of the sugar chains determined by 2D mapping method.** The N-linked sugar chains of PPL2A were analyzed by 2D mapping method and MALDI-TOF-MS. Briefly, tryptic peptides of CAM-PPL2A containing glycopeptides were separated by reversed-phase HPLC on a TSKgel ODS 120T column. N-linked sugar chains obtained from glycopeptides by digestion with glycopeptidase A (0.2 mU) were reductively aminated with 2-aminopyridine (PA). To confirm the structure of sugar chains, they were treated with a fucosidase. Then, PA-sugar derivatives were analyzed by 2D mapping method with two different columns; TSKgel ODS 120T and TSKgel Amide-80 columns, and detected by fluorescence (Ex/Em = 320/380 nm). The retention time of unknown PA-sugar was converted to glucose units, which were estimated by the elution time of standards, PA-isomaltooligosaccharide mixtures. Mr: the molecular masses of sugar chains determined by MALDI-TOF-MS. **Table S5. Multiplexed relative protein quantitation by nanoLC-TOF/TOF-MS analysis combined with iTRAQ regents.**
(DOCX)
