# Supplementary material for: Novel Matrix Proteins of Pteria penguin Pearl Oyster Shell Nacre Homologous to the Jacalin-Related β-Prism Fold Lectins
Source: PLoS One. 2014 Nov 6;9(11):e112326. doi: 10.1371/journal.pone.0112326 (PMC4223035; doi:10.1371/journal.pone.0112326)
Supplement: File S1 — Supporting information. Figure S1. Silica gel TLC profiles of sugars and polyols extracted from Pteria penguin organs. Reference sugars were maltotriose (Maltri), maltose (Mal), sucrose (Suc), trehalose (Tre), and glucose (Glc). Polyols and free sugars samples were isolated from the adductor muscle (Ad), gill (Gi), body fluid (Bf) and mantle (Ma) of Pteria penguin, respectively. +Inv indicates the invertase-reatment, which convert the sucrose to glucose and fructose. TLC was developed by ethylacetate/acetic acid/methanol/water (60∶15∶15∶10). Sugars on the plate were visualized by the orcinol-sulfuric acid method. Arrows indicate the migration of trehalose spot. Figure S2. Reverse phase HPLC of peptides generated by proteolytic digestion of CAM-PPL2s. CAM-PPL2s (α, β, and γ subunits) were digested with endoproteinase Arg-C (A), with Acromobacter protease I (B) and S. aureus V8(C), respectively. Peptides were separated by reversed-phase HPLC on a TSKgel ODS 120T column (5 µm, 4.6 Å∼250 mm) using a linear gradient increase of acetonitrile in 0.1% trifluoroacetic acid. The flow rate was 1 ml/min. Peptide maps for α subunit (upper), for βsubunit (middle), and for γ subunit (lower). Figure S3. MALDI-TOF mass spectrometry of glycopeptides derived from PPL2A α subunit before (A) and after glycopeptidase A treatment (B). Glycopeptide was digested using 0.2 mU of glycopeptidase A (Seikagaku Kogyo, Japan) and purified by reversed-phase HPLC on TSKgel ODS 120T column (4.6 mm Å∼250 mm, Tosoh), with monitoring by the phenol-sulfuric acid method. The purified sugar chains were reductively aminated with 2-aminopyridine and boranedimethylamine complex. The PA-sugar chains were analyzed using a 2D mapping method with 2 different kinds of columns; TSKgel Amide-80 column (4.6 mm Å∼250 mm, Tosoh) at a flow rate of 0.5 ml/ml at 40°C using 2 solvents, 3% acetic acid in water with triethylamine (pH 7.3) and acetonitrile (35∶65 by volume), and 3% acetic acid in water with triethylamine (pH [file pone.0112326.s001.docx]

**Supplemental figures and tables**

Naganuma *et al.*


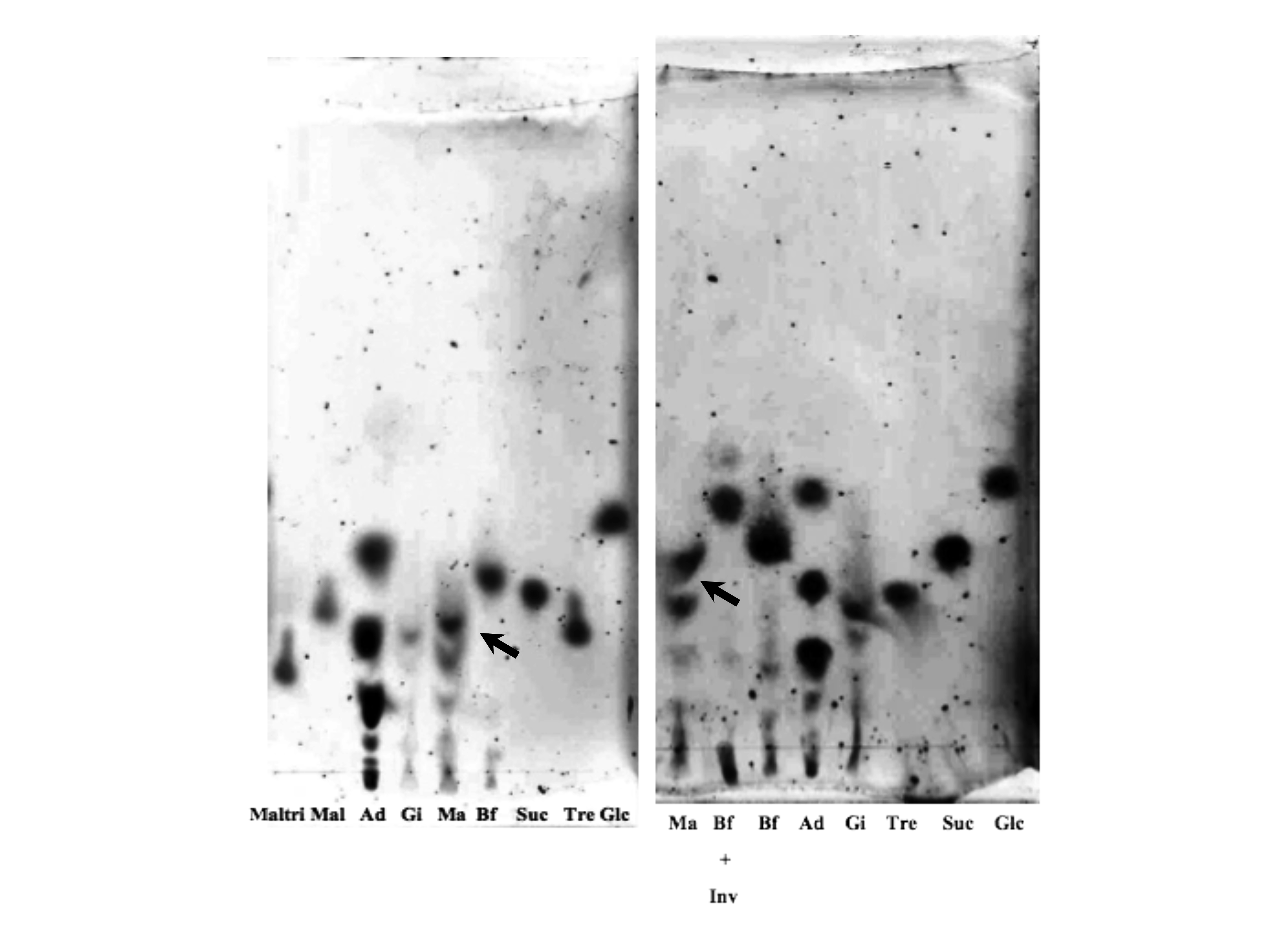


**Figure S1. Silica gel TLC profiles of sugars and polyols extracted from *Pteria penguin* organs.** Reference sugars were maltotriose (Maltri), maltose (Mal), sucrose (Suc), trehalose (Tre), and glucose (Glc). Polyols and free sugars samples were isolated from the adductor muscle (Ad), gill (Gi), body fluid (Bf) and mantle (Ma) of *Pteria penguin*, respectively. +Inv indicates the invertase-reatment, which convert the sucrose to glucose and fructose. TLC was developed by ethylacetate/acetic acid/methanol/water (60:15:15:10). Sugars on the plate were visualized by the orcinol-sulfuric acid method. Arrows indicate the migration of trehalose spot.

**
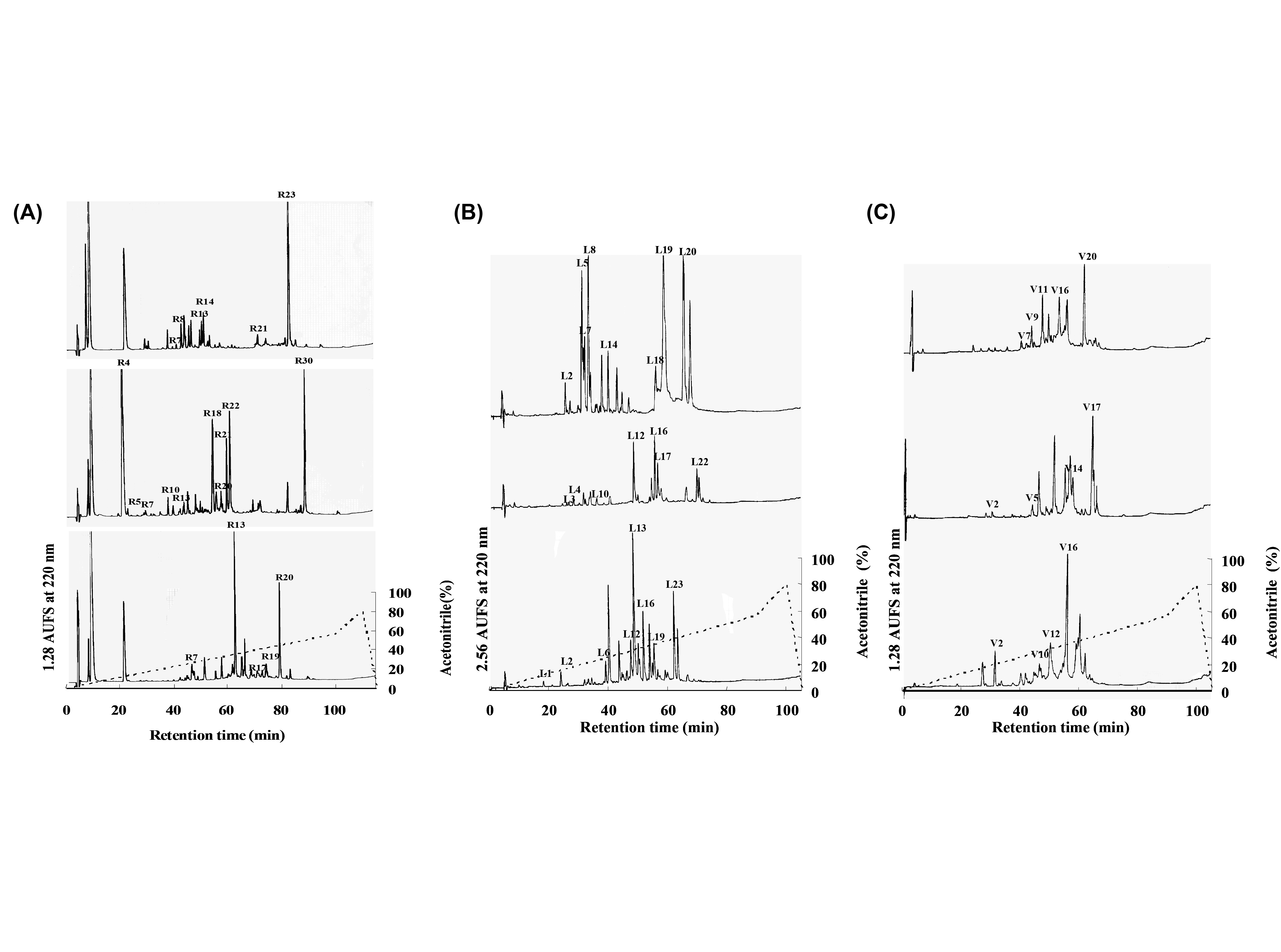
**

**Figure S2. Reverse phase HPLC of peptides generated by proteolytic digestion of CAM-PPL2s.** CAM-PPL2s (α, β, and γ subunits) were digested with endoproteinase Arg-C (A), with *Acromobacter* protease I (B) and *S. aureus* V8(C), respectively. Peptides were separated by reversed-phase HPLC on a TSKgel ODS 120T column (5 μm, 4.6 Å~ 250 mm) using a linear gradient increase of acetonitrile in 0.1% trifluoroacetic acid. The flow rate was 1 ml/min. Peptide maps for α subunit (upper), for βsubunit (middle), and for γ subunit (lower).


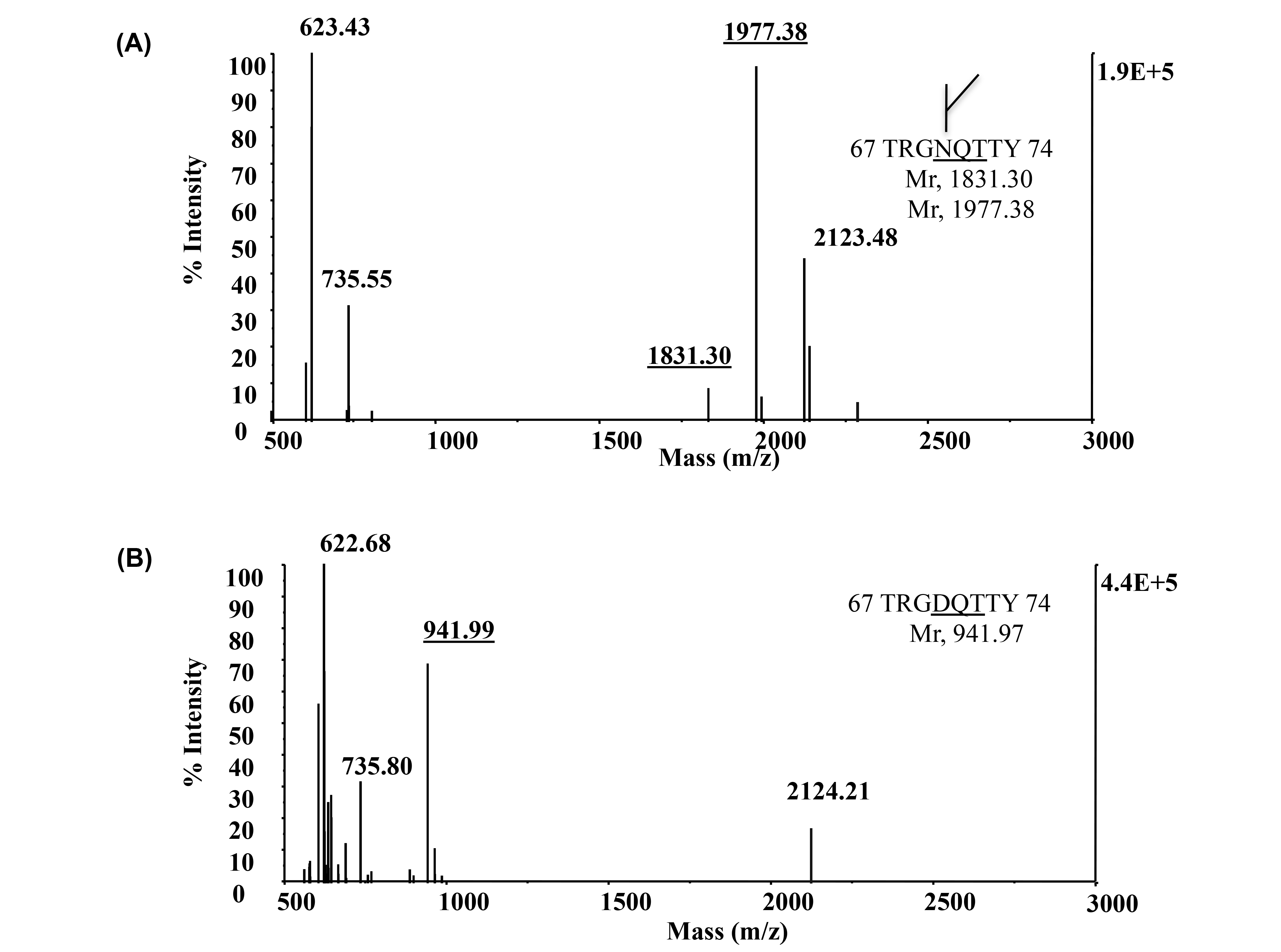


**Figure S3. MALDI-TOF mass spectrometry of glycopeptides derived from PPL2A α subunit before (A) and after glycopeptidase A treatment (B).** Glycopeptide was digested using 0.2 mU of glycopeptidase A (Seikagaku Kogyo, Japan) and purified by reversed-phase HPLC on TSKgel ODS 120T column (4.6 mm Å~ 250 mm, Tosoh), with monitoring by the phenol-sulfuric acid method. The purified sugar chains were reductively aminated with 2-aminopyridine and boranedimethylamine complex. The PA-sugar chains were analyzed using a 2D mapping method with 2 different kinds of columns; TSKgel Amide-80 column (4.6 mm Å~ 250 mm, Tosoh) at a flow rate of 0.5 ml/ml at 40°C using 2 solvents, 3% acetic acid in water with triethylamine (pH 7.3) and acetonitrile (35:65 by volume), and 3% acetic acid in water with triethylamine (pH 7.3) and acetonitrile (50:50 by volume), and detected by fluorescence (Ex/Em = 320/380 nm). The retention time of unknown PA-sugar was converted to glucose units, which were estimated by the elution time of standard, PA-isomaltooligosaccharide mixtures.


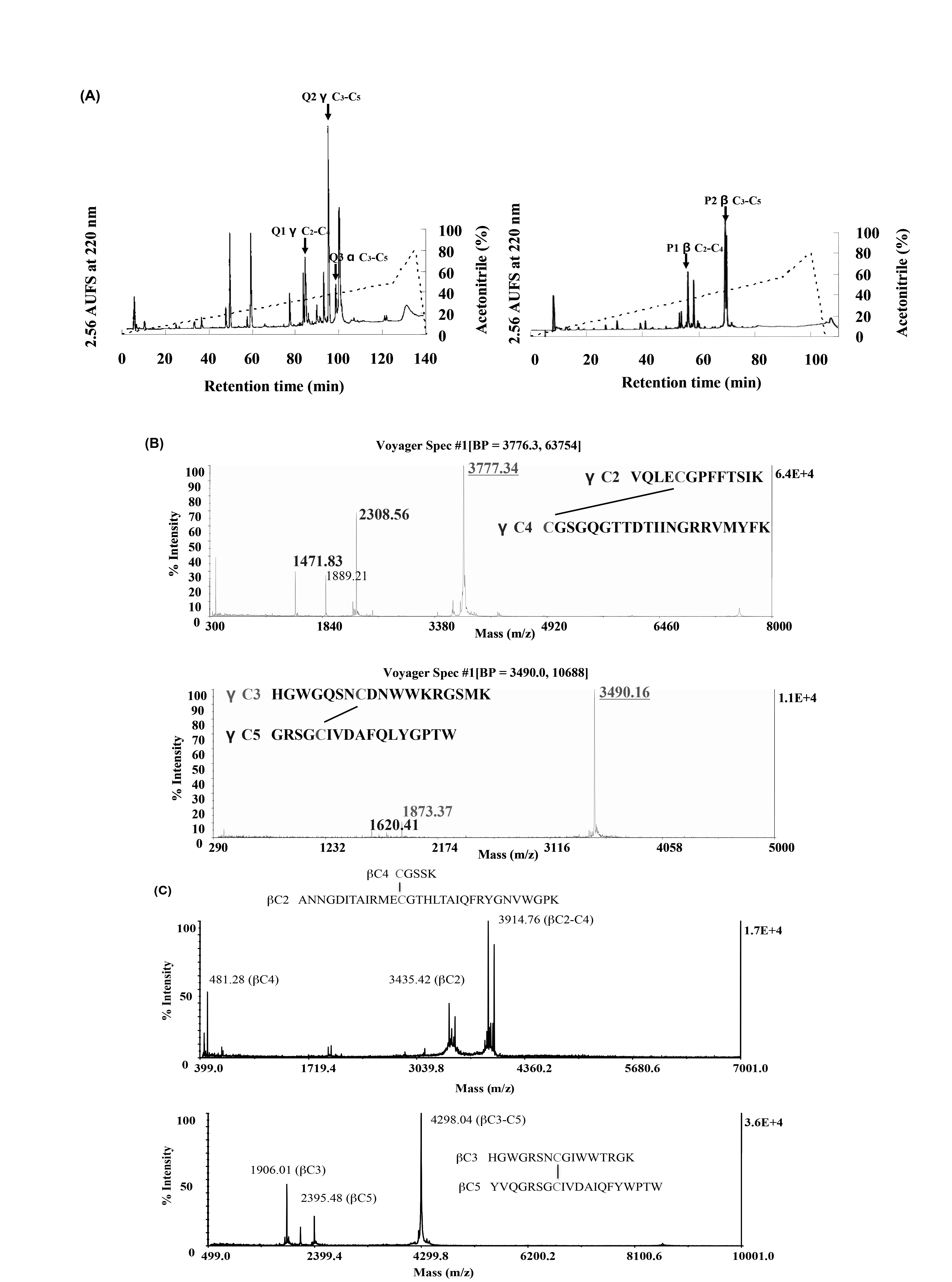


**Figure S4. Peptide mapping for determination of disulfide bonds of PPL2s.** Chromatogram of Lys-C digestion of PPL2A and PPL2B (A), and MALDI-TOF mass spectrometry of fragments including disulfide bonds derived from PPL2A (B) and 2B (C).


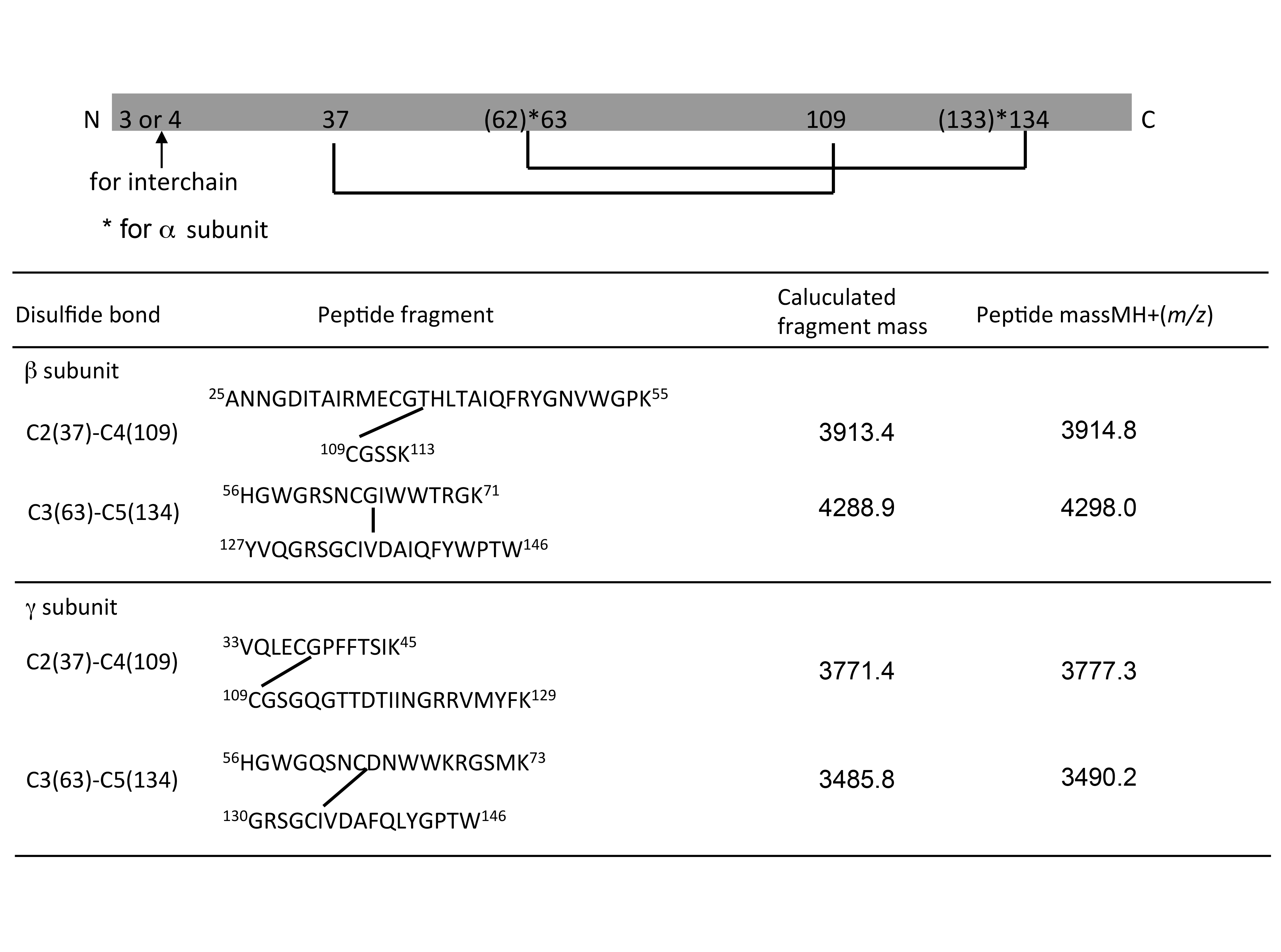


**Figure S5. Disulfide bond structures of PPL2 subunits.** Positions of the disulfide bonds in PPL2 subunits were identified by analyzing the peptide fragments, C2(37)-C4(109) and C3(63)-C5(134) for β subunit and C2(37)-C4(109) and C3(63)-C5(134) for γ subunit, respectively, which were derived from the unmodified proteins upon endoproteinase Lys-C cleavage, and subsequent analyses by a protein sequencer and MALDI-TOF mass spectrometer.


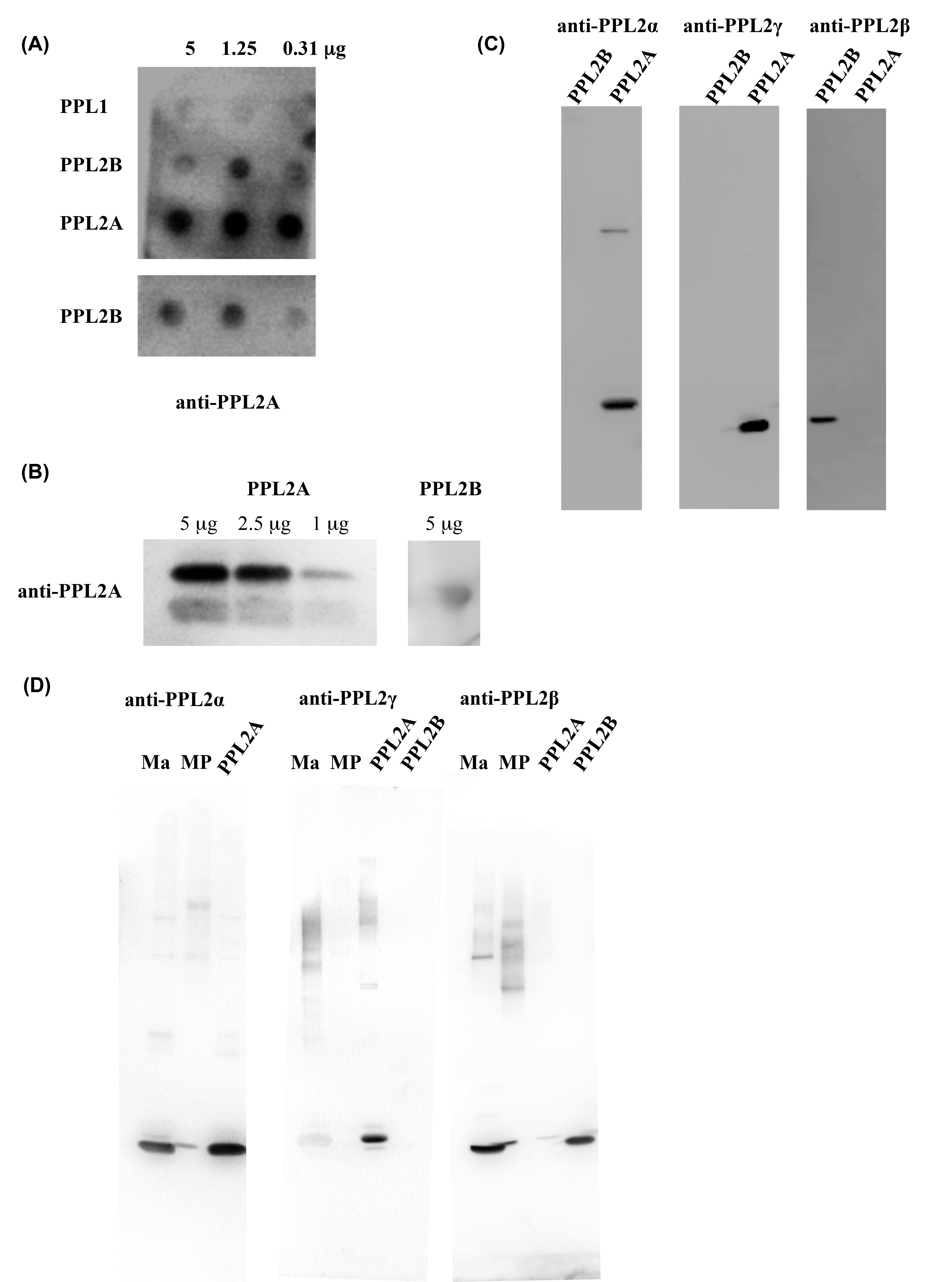


**Figure S6. Specificity of anti-PPL2A antibody checked by dot blot (A) and Western blot analyses (B), and specificities of anti-PPL2 α, β, and γsubunits antibodies (C) and Western blot profiles of anti-PPL2 subunits antibodies for the secretory fluid of mantle (Ma) and nacreous matrix proteins (MP) of *P. penguin* (D).**

**
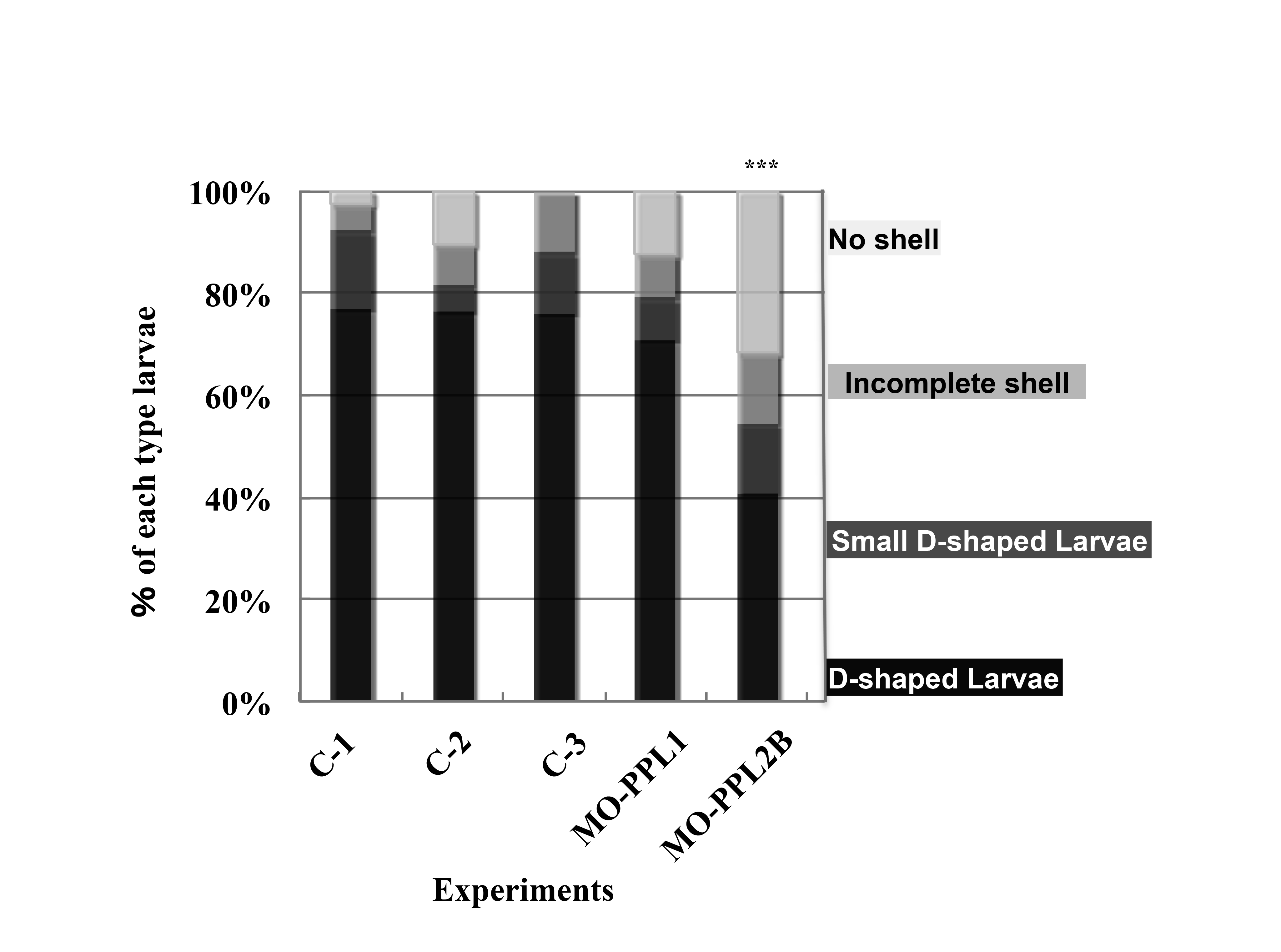
**

**Figure S7. Knockdown analysis of PPL1 and PPL2B.** Controls C-1: treated without Endo-porter, C-2: treated with Endo-porter, C-3: treated with Endo-porter and control oligos, MO-PPL1: treated with morpholino oligo for PPL1, MO-PPL2B: treated with morpholino oligo for PPL2B. Each experiment was conducted in duplicate and analyzed using chi-square tests (df=2). ***: p<0.001.

**
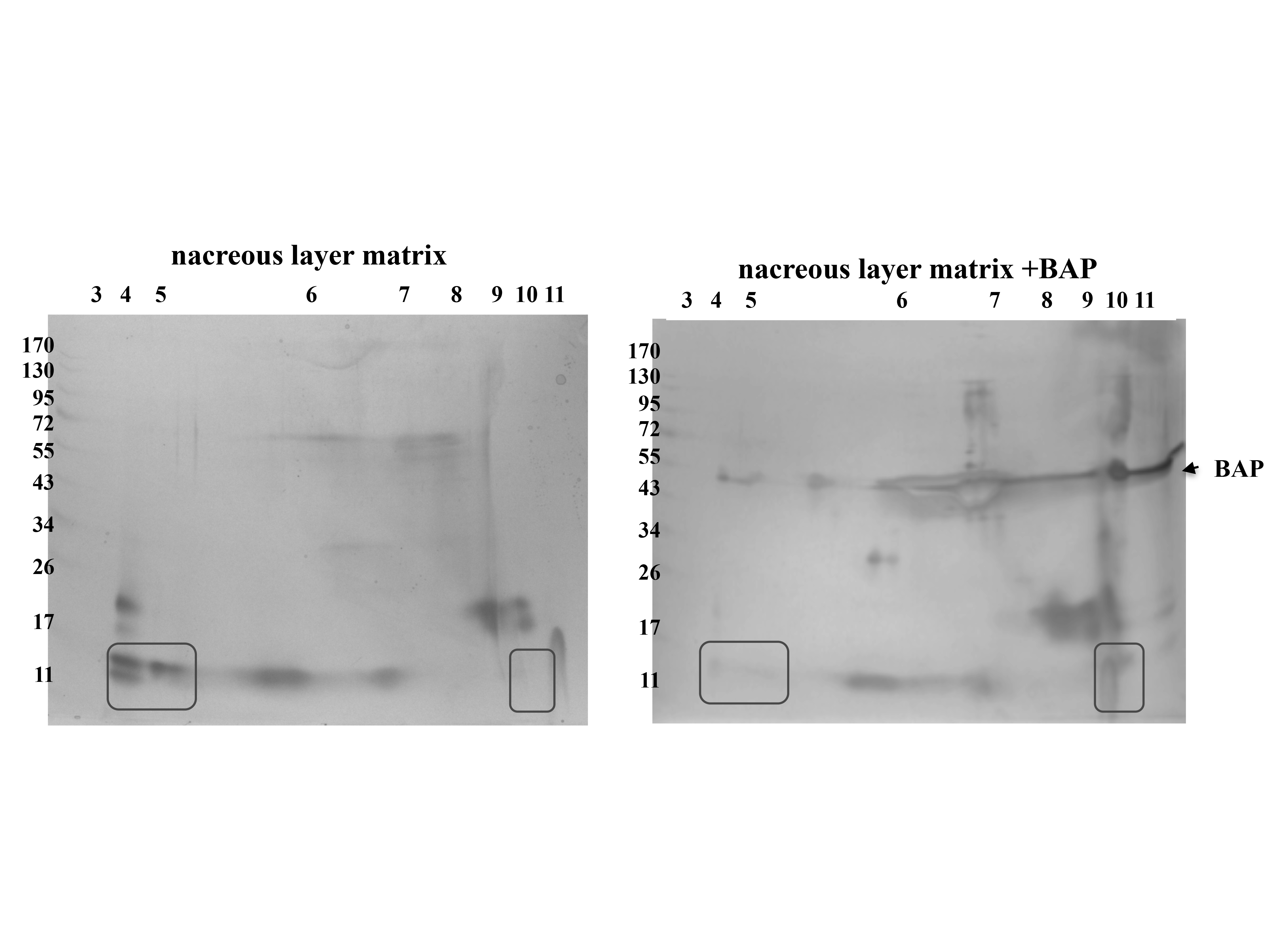
**

**Figure S8. 2D PAGE analysis of matrix proteins of *P. penguin* nacreous layer with/without dephosphorylation.**

2D PAGE analysis of nacreous matrix proteins were carried using the IPGphor, isoelectric focusing (IEF) system with IPG strips (3-11NL) and SDS-PAGE on 15% slab gels. +BAP: Dephosphorylation of nacreous matrix proteins by incubating with bacteria alkaline phosphatase (BAP, 0.6 U) at 30˚C overnight. Some acidic spots were shifted to basic corresponding to PPL2s by BAP treatment (indicated by box).

**
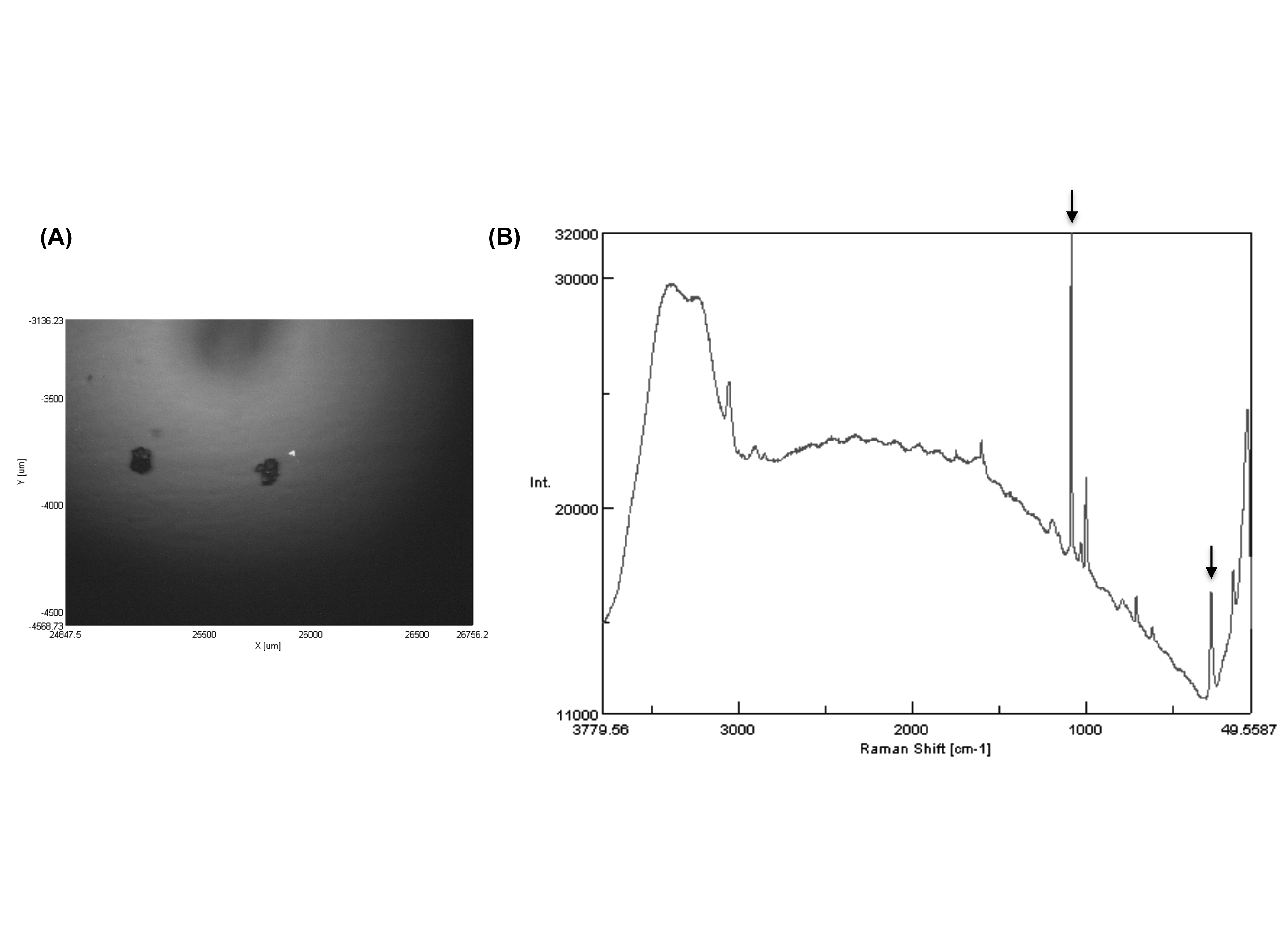
**

**Figure S9. Microscopic laser Raman spectroscopy of CaCO_3_ polycrystalline derived from PPL2A.**  Micrographs of crystals (A) and typical Raman spectrum for calcite characterized by peaks at 1090 cm^-1^ and 279 cm^-1^ (indicated by arrows) (B).

**
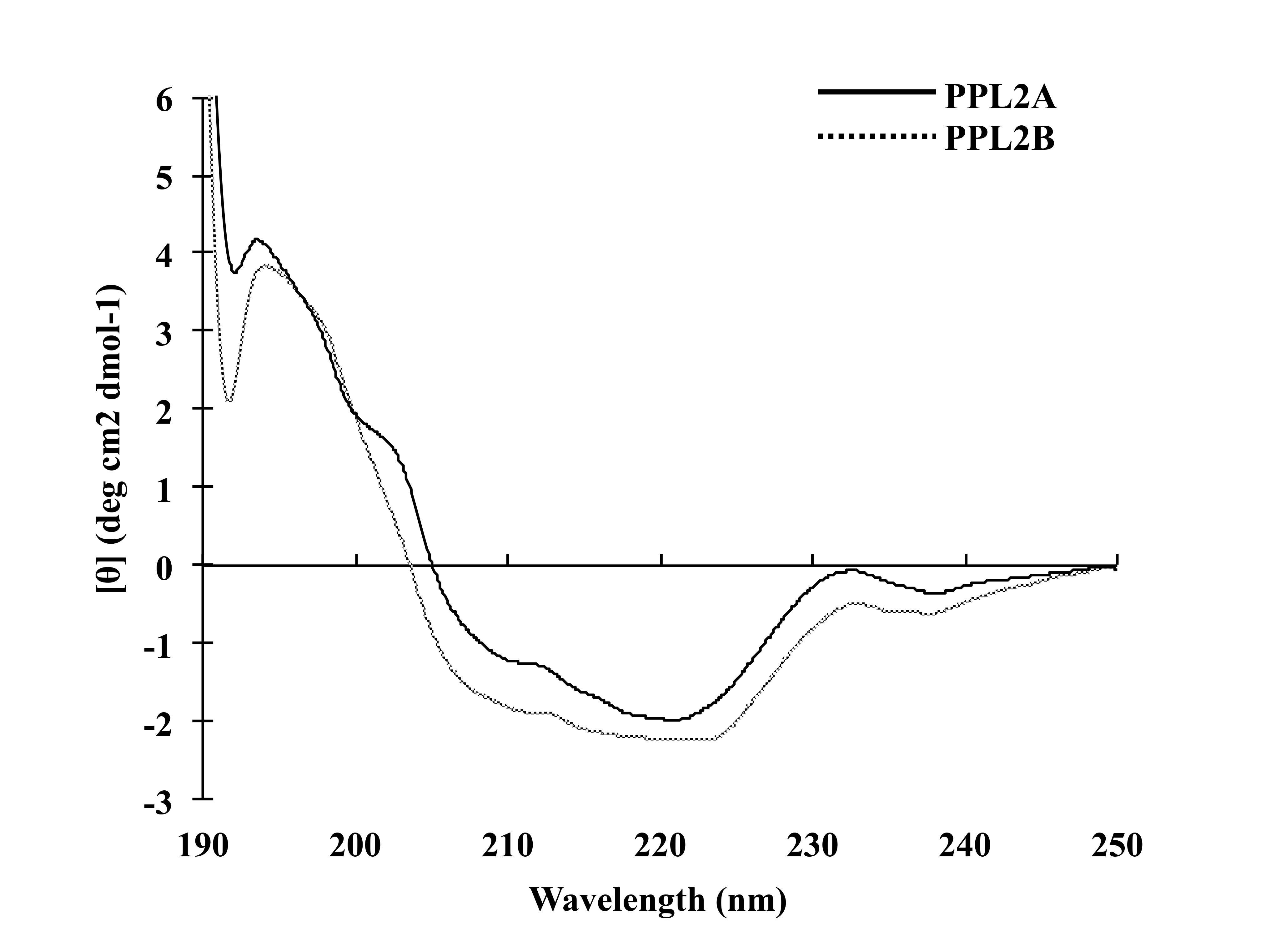
**

**Figure S10. CD spectra of PPL2A and PPL2B.**  CD spectra were measured at room temperature using a Jasco J-720 spectropolarimeter (Jasco, Tokyo, Japan) in the range of 210-260 nm with a 0.5 cm path length. Protein solutions were prepared at 0.1% (W/V) in 50 mM Tris-HCl (pH 7.5).

**Table S1. Primer sequences for 5’-, 3’- RACE and conditions of PCR** **amplification.**

**
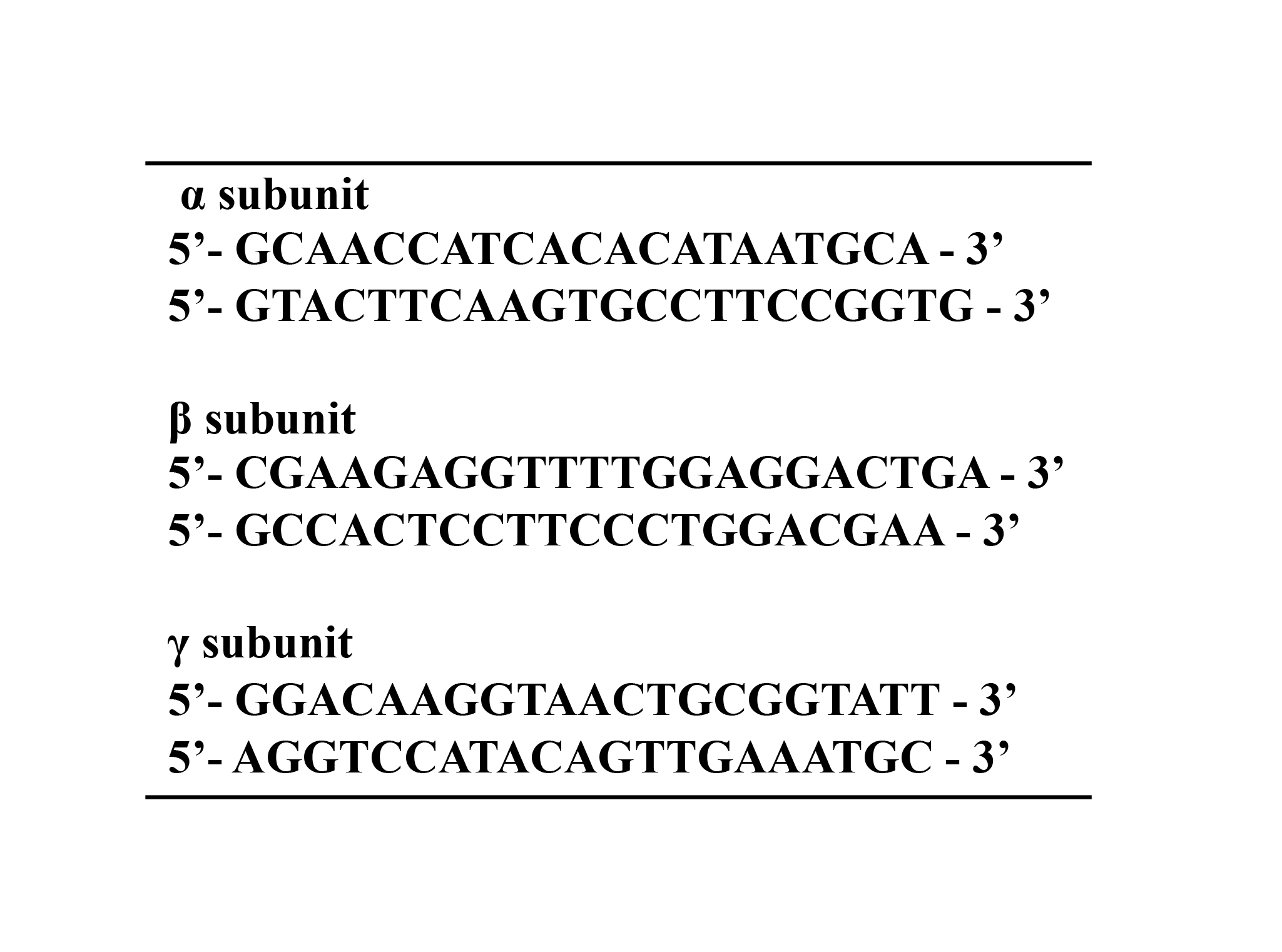
**

**Table S2. Inhibition of hemagglutination activity of PPL2s by saccharides.**

**
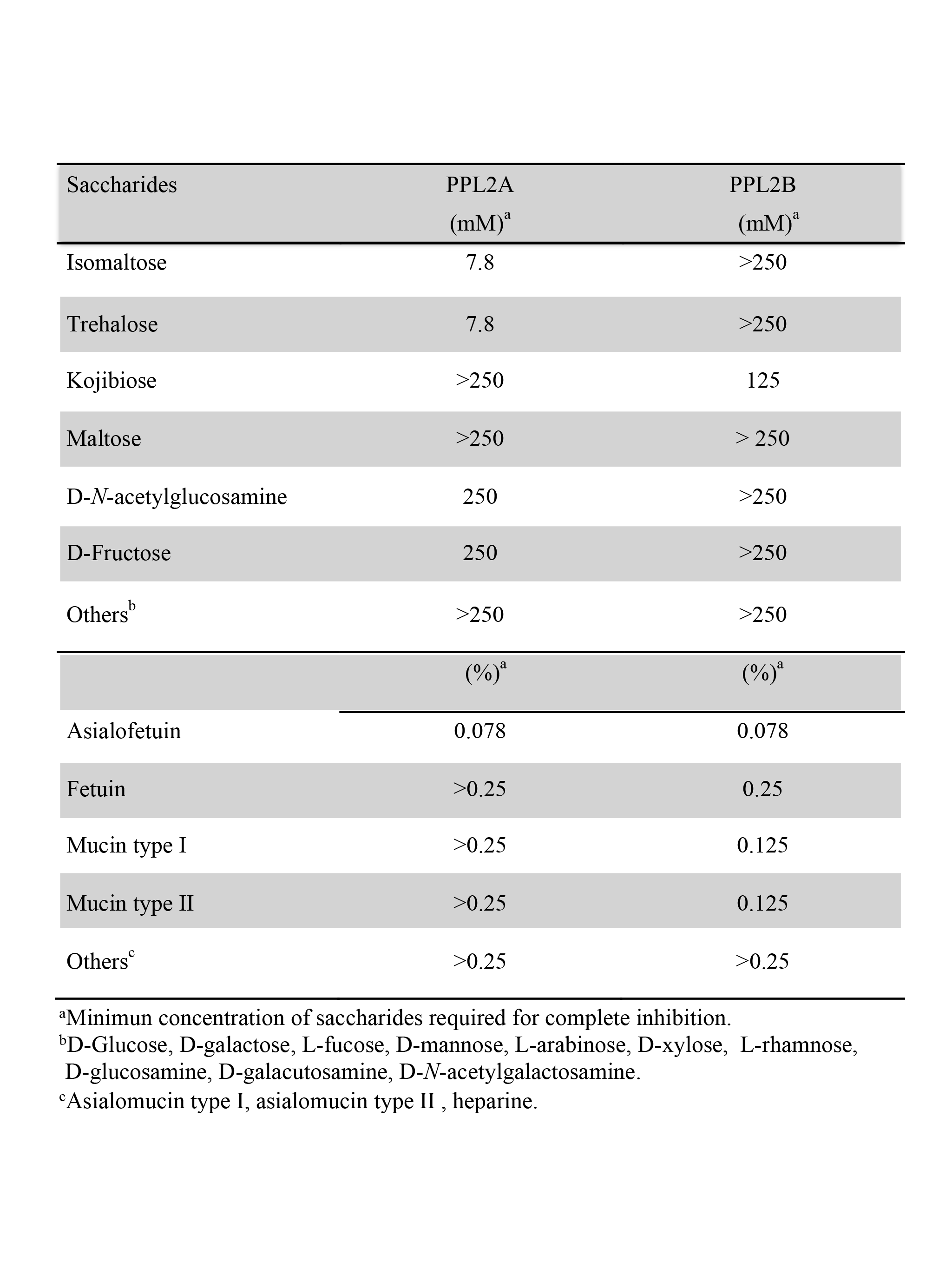
**

**Table S3. Amino acid sequences of peptide fragments derived from PPL2A (α, γ) and 2B (β).**

Amino acids sequence m/z

PPL2α Observed Calculated

L2 RNFAK 637.17 636.74

L5 CGWSTGAK 867.73 868.01

L7 STTITGRHLK 1115.92 1115.28

L8 YGSTWGTK 900.83 900.97

L14 AFCSQYLTK 1119.77 1119.33

L18 VGGNGGGAFDESSLSSNGDITAIQVECGNVFTSIK 3492.27 3490.79

L19 HGWGASHCSNYFTRGNQTTYTLEDGFYITGATITHNK 5103.44 4191.51

L20 YINGRSGCIVDSLSLYWPQWS 2504.06 2502.85

R7 YGSTWGTK 900.90 901.97

R8 HLKYINGR 1001.66 1002.17

R13 NFAKCGWSTGAKSTTITGR 2045.66 2046.33

R14 HGWGASHCSNYFTR 1679.96 1680.84

R21 GNQTTYTLEDGFYITGATITHNKYVNSIIFKTNKR 4984.61 3996.41

R23 SGCIVDSLSLYWPQWS 1900.78 1899.17

V7 VITGATITHNKYVNSIIFKTNKRNFAKCGWSTGAKSTTIT ND 6329.33

GRHLKYINGRSGCIVD

V9 SSLSSNGDITAIQVE 1524.00 1521.61

V11 AFCSQYLTKVGGNGGGAFDE 2083.67 2080.29

V16 CGNVFTSIKVKYGSTWGTKHGWGASHCSNYFTRGNQTTYTLE 5843.28 4807.37

V20 SLSLYWPQWS 1269.10 1267.42

PPL2β

L3 LPK ND 358.47

L4 GGAFTDK 696.75 696.74

L10 RFGGLK 678.71 678.82

L12 HGWGRSNCGIWWTRGK 1959.63 1960.24

L16 VSYNLAPREYISGATISYGDYVNSVRIK 3138.25 3138.51

L17 ANNGDITAIRMECGTHLTAIQFRYGNVWGPK 3494.20 3492.00

L22 YVQGRSGCIVDAIQFYWPTW 2448.62 2448.81

R4 SKSVSGKR 850.35 850.97

R5 IKTNKR 761.37 761.92

R7 KLPKCGSSKGEKSK 1539.40 1539.85

R10 LKYVQGR 865.46 865.03

R13 GKKVSYNLAPR 1234.77 1235.45

R18 YGNVWGPKHGWGR 1515.87 1515.68

R19 RFGGLKGGAFTDKSKANNGDITAIR 2439.72 2439.73

R20 MECGTHLTAIQFR 1565.69 1564.86

R21 EYISGATISYGDYVNSVR 1996.04 1995.14

R22 SNCGIWWTR 1181.58 1180.37

R30 SGCIVDAIQFYWPTW 1843.76 1844.13

V2 VRIKTNKRKLPKCGSSKGE 2186.65 2191.24

V5 YISGATISYGDYVNSVRIKTNKRKLPKCGSSKGE 1612.41 1615.71

V14 CGTHLTAIQFRYGNVWGPKHGWGRSNCGIWWTRGKKVS 5306.68 5308.12

YNLAPRE

V17 KSKSVSGKRLKYVQGRSGCIVDAIQFYWPTW 3652.31 3651.28

PPL2γ

L1 RGSMK ND 579.70

L2 SFDK ND 495.53

L6 YINSVTLTTNK 1255.15 1255.42

L12 CGSGQGTTDTIINGRRVMYFK 2363.81 2363.72

L13 HGWGQSNCDNWWK 1675.74 1676.82

L16 EYTLGANEYITGAIVSHGK 2025.24 2025.23

L19 VQLECGPFFTSIK 1527.64 1525.83

L23 GRSGCIVDAFQLYGPTW 1929.32 1929.21

R7 SFDKCGSGQGTTDTIINGR 2017.99 2016.20

R13 YGTTWAPKHGWGQSNCDNWWKR 2738.97 2739.03

R17 GSMKEYTLGANEYITGAIVSHGKYINSVTLTTNKK 3793.65 3795.30

R19 GLITKVQLECGPFFTSIKVR 2295.68 2296.38

R20 SGCIVDAFQLYGPTW 1715.00 1714.97

V2 YTLGANE 762.87 767.81

V12 YITGAIVSHGKYINSVTLTTNKKSFDKCGSGQGTTDTIING 6180.42 6181.12

RRVMYFKGRSGCIVD

V18 AFQLYGPTW 1085.49 1083.22

Peptide fragments designated L, R, and V were corresponding to the digests with *Acromobacter* protease I, endoproteinase Arg-C and *S. aureus* V8, respectively. Mass numbers (observed) of peptides were determined by MALDI-TOF MS analysis and compared with those calculated from the sequences.

**Table S4. Structures of the sugar chains determined by 2D mapping method.**

**
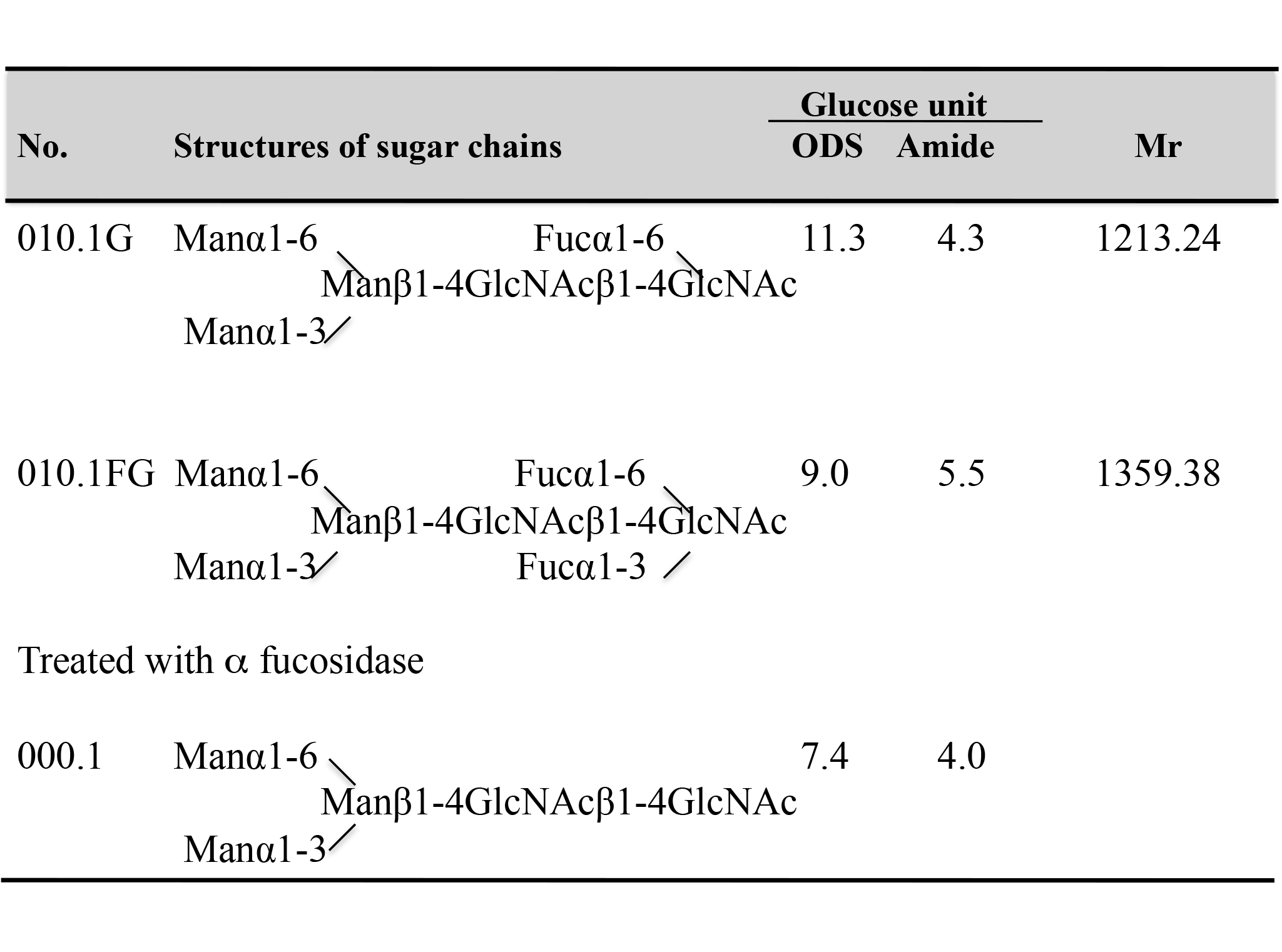
**

The N-linked sugar chains of PPL2A were analyzed by 2D mapping method and MALDI-TOF-MS. Briefly, tryptic peptides of CAM-PPL2A containing glycopeptides were separated by reversed-phase HPLC on a TSKgel ODS 120T column. N-linked sugar chains obtained from glycopeptides by digestion with glycopeptidase A (0.2 mU) were reductively aminated with 2-aminopyridine (PA). To confirm the structure of sugar chains, they were treated with a fucosidase. Then, PA-sugar derivatives were analyzed by 2D mapping method with two different columns; TSKgel ODS 120T and TSKgel Amide-80 columns, and detected by fluorescence (Ex/Em = 320/380 nm). The retention time of unknown PA-sugar was converted to glucose units, which were estimated by the elution time of standards, PA-isomaltooligosaccharide mixtures. Mr: the molecular masses of sugar chains determined by MALDI-TOF-MS.

**Table S5. Multiplexed relative protein quantitation by nanoLC-TOF/TOF-MS analysis combined with iTRAQ regents.**

**
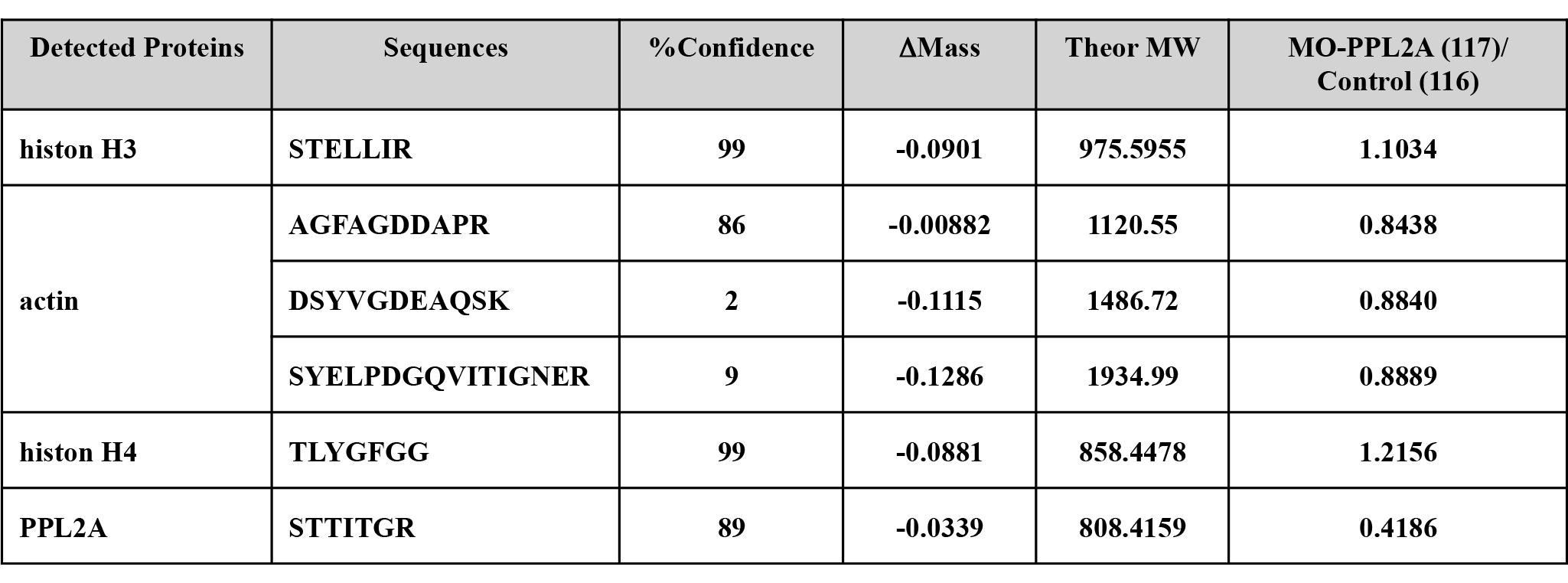
**
